# Heparanase-Dependent Remodeling of Initial Lymphatic Glycocalyx Regulates Tissue-Fluid Drainage During Acute Inflammation *in vivo*

**DOI:** 10.3389/fimmu.2019.02316

**Published:** 2019-10-04

**Authors:** Samantha Arokiasamy, Ross King, Hidayah Boulaghrasse, Robin N. Poston, Sussan Nourshargh, Wen Wang, Mathieu-Benoit Voisin

**Affiliations:** ^1^Barts and the London School of Medicine and Dentistry, William Harvey Research Institute, Queen Mary University of London, London, United Kingdom; ^2^School of Engineering and Materials Science, Institute of Bioengineering, Queen Mary University of London, London, United Kingdom

**Keywords:** glycocalyx, lymphatic vessels, neutrophil trafficking, inflammation, lectins, heparan sulfate, heparanase, lymphatic drainage

## Abstract

The glycocalyx is a dense layer of carbohydrate chains involved in numerous and fundamental biological processes, such as cellular and tissue homeostasis, inflammation and disease development. Composed of membrane-bound glycoproteins, sulfated proteoglycans and glycosaminoglycan side-chains, this structure is particularly essential for blood vascular barrier functions and leukocyte diapedesis. Interestingly, whilst the glycocalyx of blood vascular endothelium has been extensively studied, little is known about the composition and function of this glycan layer present on tissue-associated lymphatic vessels (LVs). Here, we applied confocal microscopy to characterize the composition of endothelial glycocalyx of initial lymphatic capillaries in murine cremaster muscles during homeostatic and inflamed conditions using an anti-heparan sulfate (HS) antibody and a panel of lectins recognizing different glycan moieties of the glycocalyx. Our data show the presence of HS, α-D-galactosyl moieties, α2,3-linked sialic acids and, to a lesser extent, N-Acetylglucosamine moieties. A similar expression profile was also observed for LVs of mouse and human skins. Interestingly, inflammation of mouse cremaster tissues or ear skin as induced by TNF-stimulation induced a rapid (within 16 h) remodeling of the LV glycocalyx, as observed by reduced expression of HS and galactosyl moieties, whilst levels of α2,3-linked sialic acids remains unchanged. Furthermore, whilst this response was associated with neutrophil recruitment from the blood circulation and their migration into tissue-associated LVs, specific neutrophil depletion did not impact LV glycocalyx remodeling. Mechanistically, treatment with a non-anticoagulant heparanase inhibitor suppressed LV HS degradation without impacting neutrophil migration into LVs. Interestingly however, inhibition of glycocalyx degradation reduced the capacity of initial LVs to drain interstitial fluid during acute inflammation. Collectively, our data suggest that rapid remodeling of endothelial glycocalyx of tissue-associated LVs supports drainage of fluid and macromolecules but has no role in regulating neutrophil trafficking out of inflamed tissues via initial LVs.

## Introduction

The glycocalyx is a carbohydrate-enriched layer surrounding all mammalian cells that is implicated in many biological and pathophysiological responses. *In vivo*, the glycocalyx of blood capillaries, the most studied cell surface glycan layer, spans between several 100 nm to a few micrometers on the luminal side, proportional to the size of vessels (1–3). Biochemically, this glycocalyx is composed of chains of carbohydrate residues attached to transmembrane glycoproteins, sulfated proteoglycans and to glycosaminoglycan (GAG) side chains. Among the glycoproteins forming the glycocalyx of blood vessels are cell adhesion molecules of short molecular length, such as intercellular adhesion molecules (e.g., ICAM-1/2 and VCAM-1) and selectins (4). Proteoglycans on the other hand are considered to be the “backbone” molecules of the glycocalyx (4) and are either anchored to the cell membrane (syndecans, glypicans) or secreted into the glycocalyx structure (mimecans, perlecans, and biglycans). GAG side chains are bound to these proteoglycans and are comprised of heparan sulfate (HS), chondroitin sulfate (CS), and hyaluronan (HA), with HS being the most abundant in the endothelial glycocalyx (5). GAGs are involved in numerous homeostatic and pathological functions of blood vessels through their interactions with a variety of proteins within the lumen of blood vessels. Indeed, the blood vascular endothelial cell (BEC) glycocalyx forms an integral part of the vascular barrier between flowing blood and the interstitium. As such, BEC glycocalyx plays a critical role in vascular permeability and the modulation of inflammatory processes (4, 6, 7). Specifically, the blood vascular glycocalyx acts as a mechano-transducer of sheer stress forces from the blood flow and induces the release of nitric oxide to regulate vascular tone (8). Furthermore, the sulfated GAG side-chains and the high density of glycans also provide strong negative electrostatic charges along the luminal surface of BECs that repulse red blood cells from the endothelium and restrict the diffusion of plasma proteins and solutes through the vessel wall into the interstitium. In contrast, thinning of BEC glycocalyx is strongly associated with increased vascular permeability and edema formation (9, 10).

The blood vascular glycocalyx is also intimately associated with the initial steps of leukocyte recruitment. Specifically, The BEC glycocalyx promotes the interaction between the leukocyte-expressed adhesion molecule L-selectin with its glycosylated receptor PSGL-1 on the luminal side of the endothelium. This interaction allows leukocyte integrins to access endothelial cell adhesion molecules, such as ICAM-1 and VCAM-1 during the rolling and adhesion stages of the recruitment cascade, respectively (11). Furthermore, the glycocalyx GAG side chains are known to bind, and to immobilize, leukocyte pro-inflammatory chemoattractants, in particular the neutrophil chemokines CXCL1 and CXCL2 (12). The latter promotes the transition from rolling to firm adhesion and supports directional crawling of neutrophils onto the luminal aspect of blood vessels. Similarly, HS chains present on high endothelial venules control CCL21 chemokine presentation during the recruitment of naïve lymphocytes and DCs to lymph nodes (13). Paradoxically, inflammation can modify the structure and function of blood vessel glycocalyx; and many cells including leukocytes and endothelial cells can release proteolytic enzymes and reactive oxygen/nitrogen species that degrade or modify the BEC glycocalyx. This phenomenon is particularly important for leukocyte recruitment as most of the adhesion molecules involved in neutrophil-EC interactions protrude <40 nm from the cell membrane whilst the thickness of the BEC glycocalyx is around 500 nm (14). Studies have demonstrated that inflammatory mediators, such as TNF or lipopolysaccharide (LPS) can reduce the thickness of BEC glycocalyx by at least a third (15–18), allowing leukocyte-expressed adhesion molecules to reach their binding partners on the BEC surface.

In sharp contrast, the characteristics and role of the glycocalyx on lymphatic vessels (LVs) have received little attention to date. The lymphatic vasculature is the second circulatory system of high vertebrates involved in tissue homeostasis, transport of interstitial fluid and macromolecules back into the blood circulation. The lymphatic system is characterized by a unidirectional network of vessels starting in most tissues with blind-end vessels also known as initial LVs or lymphatic capillaries. Initial LVs are composed of monolayer of oak leaf-shaped endothelial cells (LECs) with loose junctions and surrounded by a thin and discontinuous basement membrane (19). Those unique vessels drain into pre-collecting vessels, subsequently merging into large collectors and then afferent lymphatic venules that are connected to lymph nodes. Lymphatic vessels are thus crucial for immune surveillance as they contribute to the transport of antigens and trafficking of antigen-presenting cells from tissues to draining lymph nodes (dLNs). The latter provides a vital means through which adaptive immune responses are initiated during infections and vaccinations. Recently, the presence of a glycocalyx layer on the luminal side of large collecting lymphatic vessels in the rat mesentery was reported by electron microscopy on isolated vessels (20). It was proposed that the structure and composition of the glycocalyx of collecting LVs and BEC might be similar. Overall, LEC glycocalyx is believed to establish cytokine/chemokine gradients within the vessels, an effect that can aid lymphocyte rolling, maintain the homeostatic balance of the tissues, and contribute to pathogen clearance (4, 20, 21). Interestingly, lymphatic vessels express a unique receptor for the glycosaminoglycan hyaluronan, known as lymphatic vessel endothelial hyaluronan receptor 1 or LYVE-1 (22). Recently, LYVE-1 has been demonstrated to serve as a docking molecule for transmigrating dendritic cells and macrophages by binding to HA present on the surface of these leukocytes (23, 24). Despite these seminal but limited studies, there is to date insufficient insight into the exact composition of the glycocalyx of tissue-associated lymphatic capillaries *in vivo*. Moreover, little is known about the putative modifications and role of this LEC glycan layer during inflammation. To address this fundamental issue, we aimed to characterize the composition, remodeling and function of the glycocalyx of initial LVs of tissues in steady-state and inflamed conditions. This was achieved through analysis by confocal microscopy of the lymphatic vasculature in whole-mount murine cremaster muscles and ear dorsal skin as well as in human skin sections using several lectins (carbohydrate-binding proteins known to bind specific carbohydrate residues) and/or antibodies against HS and HA. We found that *in vivo*, the LV glycocalyx exhibited similarities with the glycocalyx of post-capillary venules with HS, α-D-galactosyl moieties, α2,3-linked sialic acids and N-acetylglucosamine chains being present. Interestingly, acute inflammation as induced by antigen-sensitization or TNF-stimulation resulted in the rapid remodeling of the LV glycocalyx as observed by reduced detection of HS and α-D-galactosyl moieties but not of sialic acids, a response associated with the migration of neutrophils into the lymphatic vasculature. Mechanistically, we observed that pharmacological blockade of endogenous heparanase inhibited TNF-induced HS cleavage. This inhibition of glycocalyx remodeling was associated with a reduced capacity of initial lymphatic capillaries to remove fluids out of the inflamed interstitium whilst neutrophil interactions with LVs were not affected. Collectively, our findings present a novel paradigm for the role and function of initial lymphatic glycocalyx, and demonstrate that its remodeling is important for the rapid drainage of inflamed tissues but not neutrophil recruitment to the lymphatic system *in vivo*.

## Materials and Methods

### Reagents

Recombinant murine TNF was purchased from R&D Systems (Abingdon, UK), Complete Freund's Adjuvant (CFA) from AMSbio (Abingdon, UK), Ovalbumin and Evans blue from Sigma-Aldrich (Poole, UK). The following primary antibodies were used for immunofluorescence labeling for confocal imaging: rat anti–mouse LYVE-1 mAb (clone ALY7; Thermofisher, Hatfield, UK); rabbit anti-human LYVE-1 Ab (polyclonal PA1-16635, Thermofisher), non-blocking rat anti-mouse CD31 mAb (clone C390, Thermofisher); rat anti-mouse/human HEV mAb (clone MECA79, Thermofisher), rat anti-mouse GR1 mAb (clone RB6-8C5, Thermofisher), rat anti-mouse CD144 mAb (VE-Cadherin, clone BV14, Thermofisher), rat anti-mouse MRP14 mAb (clone 2B10; a gift from N. Hogg, Cancer Research UK, London, UK), rat anti-mouse F4/80 mAb (clone BM8, Biolegend, London, UK), rat anti-mouse CD115 mAb (clone AFS98, Biolegend), mouse anti-heparan sulfate (HS) mAb (10E4 epitope, clone F58-10E4, AMSbio), rabbit anti-hyaluronic acid Ab (polyclonal ab53842, Abcam, Cambridge, UK) and rabbit anti-mouse/human heparanase I Ab (polyclonal ab85543, Abcam). Isolectin-B4 (IB4), *Maackia amurensis* Lectin-1 (MAL-1), *Sambucus nigra* Agglutinin (SNA) and succinylated wheat germ agglutinin (sWGA) and their respective inhibitor carbohydrates (Galactose, lactose, N-acetylglucosamine) were purchased from Vector Labs (Peterborough, UK). All antibodies and lectins were fluorescently labeled using Alexa-fluor protein labeling kits as per manufacturer's recommendations (ThermoFisher Scientific, Paisley, UK). The non-anticoagulant heparanase inhibitor N-desulfated/re-N-acetylated heparin (NAH) was sourced from Iduron (Alderley Edge, UK).

### Animals, Treatment and Induction of Tissue Inflammation

All experiments were approved by the local biological service unit Ethical Committee at Queen Mary University of London and carried out under the Home Office Project Licenses (70/7884 and P873F4263) according to the guidelines of the United Kingdom Animals Scientific Procedures Act (1986). Wild-type C57BL/6 male mice (8–12 weeks, Charles Rivers Margate, UK) were anesthetized with isofluorane and the cremaster muscles were stimulated for up to 16 h via intrascrotal (i.s.) injection of TNF (300 ng in 300 μl of PBS) or an emulsion (50:50, 300 μl per mouse) of CFA (200 μg) with ovalbumin (200 μg). Control mice were injected with 300 μl of PBS. To induce ear inflammation, anesthetized animals received a subcutaneous (s.c.) injection of 300 ng/30 μL of TNF (or PBS as control) in the dorsal ear skin for 16 h. To inhibit heparanase activity, the non-anticoagulant heparanase inhibitor N-desulfated/re-N-acetylated heparin (NAH) was injected locally (30 ug/mouse, i.s.) 3 h after the injection of TNF. For neutrophil depletion experiments, mice were injected intraperitoneally (i.p.) with 25 μg/mouse/day of anti-GR1 antibody for 3 days preceding the induction of the inflammatory response. This technique, developed in our lab (25) leads to a specific depletion of neutrophils (>99%) but not inflammatory monocytes from the blood circulation (Supplementary Figure 3). At the end of all *in vivo* experiments, animals were humanely killed by cervical dislocation in accordance with UK Home Office regulations and the tissues were removed for subsequent analysis.

### Fluorescent Staining of Whole-Mount Murine Tissues

#### Cremaster Tissues

The labeling of blood and lymphatic vessels of the cremaster muscles *in vivo* was achieved as previously described (26). Briefly, the animals received an i.s. injection of the non-blocking dose of a fluorescently-labeled anti-LYVE-1 mAb (2 μg/mouse, Alexa555 conjugated) and/or a fluorescently-labeled non-blocking anti-CD31 mAb (2 μg/mouse, Alexa488, Alexa555, or Alexa647 conjugated depending on the antibody combination) 90 min to 2 h before the end of the inflammatory period to label the lymphatic and blood vasculatures, respectively. To label the glycocalyx sugar residues and HS, the animals also received an i.s. injection of 2 μg/animal of fluorescently labeled (Alexa647) lectins, anti-HS or anti-HA mAbs (both Alexa488 conjugated) 2 h prior to sacrificing the animals. To investigate neutrophil migration responses across post-capillary venules and migration into initial lymphatic vessels of the cremaster muscles, tissues were fixed in 4% PFA in PBS for 1 h at 4°C, then blocked and permeabilized in PBS (containing 12.5% goat serum, 12.5% fetal bovine serum [FBS] and 0.5% Triton X-100) for 4 h at room temperature. To visualize neutrophils, macrophages or VE-Cadherin, tissues were incubated with Alexa647 conjugated anti-MRP14 (0.25 μg), Alexa647 conjugated anti-F4/80 (1 μg) or Alexa647 conjugated anti-CD144 (1 μg) mAbs, respectively, in 200 μl of PBS (with 10% FBS) per pair of cremaster tissues overnight or up to 48 h at 4°C. To visualize Heparanase I, tissues were incubated with 2 μg of anti-Heparanase I mAb in 200 μl of PBS (with 10% FBS) per pair of cremaster tissues for 48 h at 4°C post-fixation and permeabilization. After three washes in PBS (30 min each), tissues were incubated with an Alexa488 conjugated goat anti-rabbit secondary mAb (Thermofisher) for 4 h. For all immunostaining procedures, tissues where washed in PBS thrice for a minimum of 30 min per wash prior to the visualization of the samples by confocal microscopy.

#### Lymph Nodes

To quantify neutrophil infiltration of the cremaster draining lymph nodes, the tissues were harvested and fixed in 4% PFA in PBS for 24 h at 4°C, then halved, blocked and permeabilized in PBS (containing 12.5% goat serum, 12.5% fetal bovine serum [FBS] and 0.5% Triton X-100) for 4 h at room temperature. Tissues were then fluorescently immunostained for neutrophils (0.25 μg/100 μl anti-MRP14 mAb, Alexa647 conjugated), high endothelial venules (HEV, 0.25 μg/100 μl of anti-HEV mAb, Alexa488 conjugated) and capsula/trabeculae (0.25 μg/100 μl of anti-LYVE-1 mAb, Alexa555 conjugated), in PBS (with 10% FBS) overnight at 4°C prior to the visualization of the tissues by confocal microscopy.

#### Ear Skin

Ears were harvested and fixed in 4% PFA for 1 h at 4°C. The two ear flaps were then separated and the skin was blocked and permeabilized in PBS (containing 12.5% goat serum, 12.5% fetal bovine serum and 0.5% Triton X-100) for 4 h at room temperature. Tissues were then fluorescently incubated with 0.25 μg of Alexa488 conjugated anti-MRP14 mAb, 1 μg of Alexa647 conjugated lectin (IB4, MAL-1 or sWGA) and 1 μg of Alexa555 conjugated anti-LYVE-1 mAb in 200 μL per ear in PBS (with 10% FBS) overnight at 4°C prior to the visualization of the tissue by confocal microscopy.

### Fluorescent Staining of Human Skin Sections

Paraffin sections of four human breast skin samples from breast carcinoma patients (6 μm thick sections) were obtained from the Breast Cancer Now Tissue Bank at the Barts Cancer Institute, QMUL, with ethical permission. The samples were from distal from breast carcinomas. Hematoxylin and eosin pre-staining from an independent pathologist confirmed the absence of tumor cells in our samples with low to moderate levels of perivascular lymphocytic infiltration. Sections were dewaxed in 2× xylene and 2× 100% ethanol baths for 5 min each, and antigen retrieval was performed at pH 9 in a citrate buffer (Vectorlabs) for 30 min at 95°C following the antibody supplier's guidelines. The sections were then blocked at room temperature with 10% FBS in PBS for 30 min followed by a 48 h incubation at 4°C with the rabbit anti-LYVE-1 antibody (1/200), anti-HS antibody (1/200) or one of our lectins (IB4, MAL-1, or sWGA) in PBS with 0.5% FBS. Samples were washed thrice with PBS for 15 min prior to visualization by confocal microscopy.

### Confocal Microscopy and Image Analysis

All samples were imaged with either a Leica SP5 or Leica SP8 confocal microscope (Leica microsystem, Milton Keynes, UK) with the use of a 20× water-dipping objective (NA:1.0). Three-dimensional confocal images from mouse and human samples were analyzed using IMARIS software (Bitplane, Zurich, Switzerland) or FIJI/Image J (NIH, Bethesda, USA).

#### Mouse Cremaster Muscles and Ear Skin

Images of lymphatic initial capillary vessels (LVs) and blood post-capillary venules (BVs) (~5 vessels per tissue) were acquired using sequential scanning of different channels at every 0.52 μm of tissue depth at a resolution of 1,024 × 470 and 1,024 × 800 pixels in the x × y plane, respectively. This resolution of pixels correspond to a voxel size of 0.45 × 0.45 × 0.52 μm in x × y × z. BV and LV were imaged at a zoom factor of ×1.9 and ×1.2, respectively. On average, a serial stack of ~60 and ~150 optical sections were acquired for BV and LV images, respectively. To assess the expression (i.e., mean fluorescence intensity measurements) of glycocalyx components, image settings (laser power, detector gain and offset) were first defined (and kept constant for each specific molecules and treatment groups) using samples stained with a control isotype-matched antibody (e.g., HS) or a lectin of interest pre-incubated with an inhibitory carbohydrate. To inhibit IB4, MAL-1 or sWGA binding activity, the lectins were pre-incubated with 50 mM of galactose, lactose or N-Acetylglucosamine, respectively, for 1 h prior to their use in murine tissues (Supplementary Figure 1). Quantification of neutrophil extravasation and migration into tissue-associated LVs were analyzed with IMARIS software as previously described (26). Specifically, extravasated neutrophils were defined as the number of neutrophils present in the interstitium across a 300 μm blood vessel segment and within 50 μm from each side of the venule of interest; and data are expressed as the number of neutrophils per volume of tissue. The neutrophil intravasation response was defined as the number of neutrophils present inside the lymphatic vessels as visualized in 3D and quantified by IMARIS Software and data were expressed as cell number per given volume of LV. LV volume were quantified by the IMARIS software following the creation of a 3D-isosurface on the LYVE-1 channel (thus excluding MRP14^+^ and CD31^high^ regions) and mean fluorescent intensity measurements of the glycocalyx components associated exclusively with LV where quantified within this isosurface. A similar strategy was done for the blood vessels (i.e., isosurface only on CD31^high^ regions).

#### Lymph Nodes

Images (12 images per pair of LNs per mouse) were obtained with the use of sequential scanning of different channels at every 0.7 μm of tissue depth at a resolution of 1,024 × 1,024 pixels in the x × y plane and with a zoom factor of 0.75, corresponding to a voxel size of 0.91 × 0.91 × 0.7 μm in x × y × z. On average, a serial stack of ~30 optical sections were acquired. Quantification of neutrophil recruitment into the dLNs were analyzed with the 3D-reconstructing image processing software IMARIS. Recruited neutrophils were defined as the number of neutrophils per volume of tissue, excluding the blood circulating neutrophils present in HEVs.

#### Human Skin Sections

Images of lymphatic vessels (LVs) were acquired using sequential scanning of different channels at every 0.52 μm of tissue depth at a resolution of 1,024 × 300 pixels in the x × y plane with a zoom factor of 1, respectively with a resolution of 0.45 × 0.45 μm in x × y plans. On average, a serial stack of ~30 optical sections were acquired, overlapping the 6 μm thick tissue section. Lymphatic vessels were confirmed by the presence of LYVE-1^+^ vessels, morphology and the absence of erythrocytes in the lumen. The expression of lectin-binding moiety and HS on LVs was analyzed by FIJI/Image J by delimitating a surface area around LYVE-1^+^ regions of LVs.

### Lymphatic Drainage Analysis

To visualize the drainage capability of lymphatic vessels of the mouse cremaster muscle *in vivo*, animals received an i.s. injection of Evans Blue in PBS (1%, 300 μL) 20 min prior to the end of the inflammatory period. The cremaster muscles, draining (inguinal) and non-draining (brachial) LNs were then harvested, snap frozen in liquid nitrogen and the blood of the animal was recovered by cardiac puncture. The blood was then centrifuged and plasma collected. For the cremaster muscles and LNs, tissues were incubated in 100% formamide at 56°C overnight prior to spectrophotometry analysis. The quantity of Evans Blue in plasma and tissues samples was quantified with a spectrophotometer at an absorbance wavelength of 620 nm.

### Blood Vascular Leakage Analysis

Blood vascular leakage (and lymphatic drainage) was assessed using the Mile's Assay (27). Briefly, 2 h before the end of the TNF stimulation (i.e., 16 h), Evans blue dye (0.5% in PBS, 5 μl/g) was injected i.v. At the end of the experiment, animals were sacrificed, and the cremaster muscles were harvested and incubated in 100% formamide (Sigma-Aldrich) at 56°C for 24 h. The amount of accumulated Evans blue in the tissue supernatant was then quantified by spectroscopy at 620 nm.

### Statistical Analysis

Data are presented as mean ± S.E.M per mouse. Significant differences between multiple groups were identified by one-way or two-way analysis of variance (ANOVA), followed by Newman-Keuls/Sidak's Multiple Comparison Test. Whenever two groups were compared, Student's *t*-test was used. *P*-values < 0.05 were considered significant.

## Results

### Characterization of the Glycocalyx of Initial Lymphatic Capillaries *in vivo*

**Figure 1:**
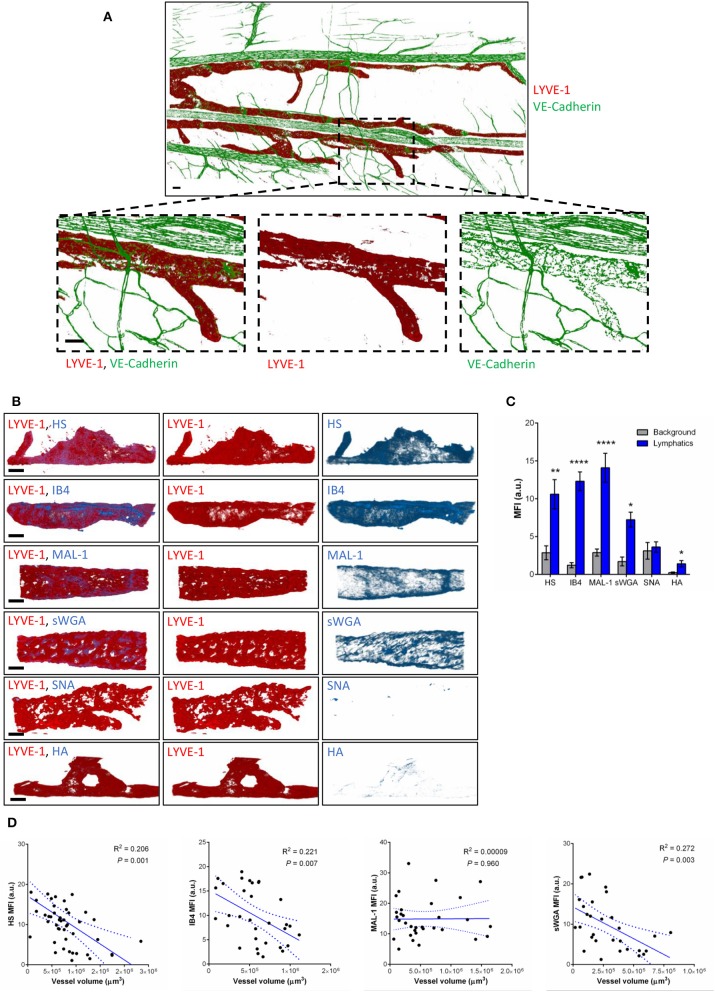
Molecular composition of the glycocalyx on initial lymphatics of mouse cremaster muscles. **(A)** Representative 3D-reconstructed confocal tiled images showing the extent of the lymphatic vasculature (LYVE-1, red) in the cremaster muscles. Bottom panels are magnified images of a region within the tissue (dotted box) demonstrating the discontinuous expression of VE-Cadherin junctions (green) in LVs as compared to blood vessels. This lymphatic organization of junctions is characteristic of lymphatic endothelial cells from initial lymphatic capillaries. **(B)** Representative 3D-reconstructed confocal images of initial lymphatic vessel (LYVE-1, red) segments and their associated staining for several glycan chains. The images show that lymphatic glycocalyx contains HS (anti-HS Ab), α-D-Galactosyl moieties (IB4), sialic acid α2,3-linked (MAL-1) glycans, and N-Acetylglucosamine moieties (sWGA), whilst sialic acid α2,6-linked glycans (SNA), or hyaluronan (HA) minimally detected. **(C)** Quantification of the mean fluorescence intensity of HS, IB4, MAL-1, sWGA, SNA, HA as quantified on the lymphatic vessel using IMARIS software. **(D)** Linear relationship between the mean vessel size and the mean fluorescence intensity of multiple glycocalyx binding proteins (anti-HS Ab, IB4, MAL-1, and sWGA). Each point represents an individual vessel from cremaster muscles. Dotted curved lines in the correlation plots represent 95% confidence interval. Bar = 50 μm. Images are representative pictures from at least 8–10 vessels/animals, with at least 5 animals per group. Significant differences between lymphatic MFI and background MFI are indicated by asterisks: *P < 0.05, **P < 0.01, ****P < 0.0001.

**Figure 2:**
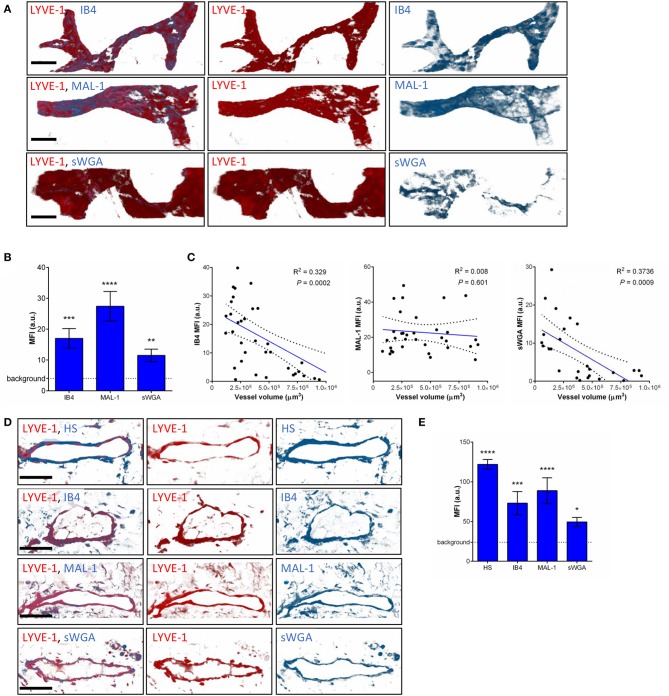
Molecular composition of the glycocalyx on initial lymphatics of mouse and human skin. **(A)** The pictures are representative 3D-reconstructed confocal images of an initial lymphatic vessel (LYVE-1, red) segment from whole-mount fixed ears of naïve mice and fluorescently labeled with IB4, MAL-1 and sWGA lectins to visualize α-D-Galactosyl, sialic acid α2,3-linked and N-Acetylglucosamine glycan moieties, respectively. **(B)** Quantification of the mean fluorescence intensity of IB4, MAL-1 and sWGA as quantified on initial lymphatic vessels of mouse ear skins. **(C)** The graphs show the linear relationship between the mean vessel size and the mean fluorescence intensity (MFI) of individual lectins (IB4, MAL-1, and sWGA). Each point represents an individual vessel from cremaster muscles. Dotted curved lines in the correlation plots represent 95% confidence interval. **(D)** The pictures are representative 3D-reconstructed confocal images of a lymphatic vessel (LYVE-1, red) from human breast skin sections and fluorescently labeled with anti-HS Ab, IB4, MAL-1, and sWGA lectins to visualize HS, α-D-Galactosyl, sialic acid α2,3-linked and N-glucosamine glycan moieties, respectively. **(E)** Quantification of the mean fluorescence intensity (MFI) of anti-HS Ab, IB4, MAL-1, and sWGA as quantified on the lymphatic vessels of human skin samples. Bar = 50 μm. Images are representative pictures from at least 8–10 vessels/sample, with at least 5 animals/4 human samples per group. Significant differences between lymphatic vessel MFI and background MFI are indicated by asterisks: *P < 0.05, **P < 0.01, ***P < 0.001, ****P < 0.0001.

**Figure 3:**
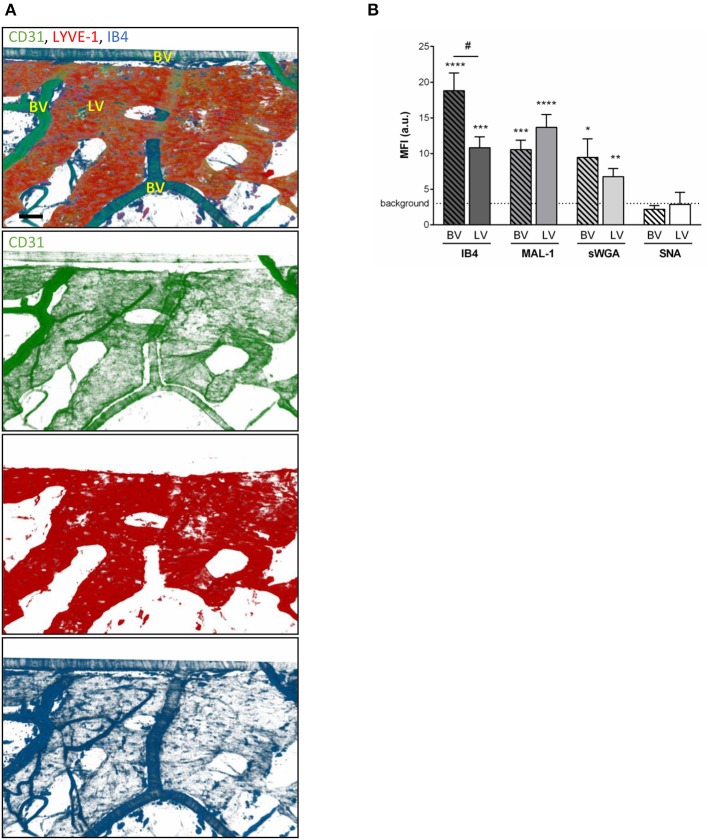
Comparison of the glycocalyx composition between initial lymphatics and blood vessels. Cremaster muscles from WT mice were whole-mount immunostained for the visualization of endothelial cells (anti-CD31 Ab, green), lymphatic vessels (anti-LYVE-1 Ab, red) and glycosylic chains from glycocalyx (lectin, blue) via i.s. injection of the antibodies/lectin for 2 h prior to being observed by confocal microscopy. **(A)** Representative 3D-reconstructed confocal images of a region of the tissue showing that IB4 binds to the blood vessels (BV) and lymphatics (LVs). (Bar = 100 um). **(B)** Quantification of the mean fluorescence intensity (MFI) for IB4, MAL-1, sWGA, and SNA binding to the surface of initial lymphatics and blood vessels as detailed in the Material and Methods. Data are expressed as mean ± SEM from at least 8–10 vessels/animal, with at least 5 animals per group. Significant differences between BV/LV MFI and background MFI are indicated by asterisks: *P < 0.05, **P < 0.01, ***P < 0.001, ****P < 0.001; and between BV and LV groups by hash symbol: ^#^*P* < 0.05.

To compensate for the lack of knowledge regarding the composition and role of initial lymphatic vessel (LV) glycocalyx, in this study we first aimed to investigate the expression profile of carbohydrate moieties present on initial LV of mouse cremaster tissues. This thin and transparent muscle contains an extensive lymphatic vasculature amenable for whole-mount fluorescent staining and 3-dimensional visualization of cellular and molecular structures *in vivo* by confocal microscopy (Figure 1A) (26). Interestingly, cremaster LVs are composed mainly of lymphatic endothelial cells (LECs) with discontinuous junctions organized in flaps and VE-Cadherin-enriched buttons (Figure 1A) (19). To visualize the glycocalyx of those initial lymphatic vessels we used several fluorescently-labeled lectins, namely Isolectin-B4 (IB4), Maackia Amurensis Lectin-1 (MAL-1), Sambucus Nigra Agglutinin (SNA) and succinylated Wheat Germ Agglutinin (sWGA). These lectins specifically recognize α-D-galactosyl moieties (IB4), sialic acid α2,3-linked (MAL-1) or α-2,6-linked (SNA) galactose/N-acetylgalactosamine residues and N-acetylglucosamine chains (sWGA). Additionally, an anti-heparan sulfate (HS) or anti-hyaluronic acid (HA) monoclonal antibody was employed to visualize heparan sulfate chains and hyaluronan, respectively. Lectins and anti-HS/anti-HA antibodies were injected i.s. in conjunction with non-blocking and fluorescently-labeled anti-LYVE-1 and anti-CD31 mAbs to differentiate the lymphatic and blood vasculatures, respectively, as described previously (26). Animals were sacrificed ~2 h later, and the cremaster muscles were removed and fixed prior to visualization and image acquisition (whole mount) by confocal microscopy. Image series were then analyzed in 3-dimensions using IMARIS software to quantify the fluorescence intensity for each specific marker binding the glycocalyx moieties associated exclusively with LVs (i.e., LYVE-1^+^ vessels). The specificity of lectin/antibody binding to their respective glycocalyx moieties in whole-mount tissues was confirmed using competitive sugar binding assays (with the use of specific inhibitory sugars for each lectins) or with isotype-matched control antibodies, respectively (Supplementary Figure 1). Our data show that *in vivo* the glycocalyx of initial lymphatic capillaries from cremaster tissues contains HS, α-D-galactosyl moieties, α-2,3-linked sialic acid residues and N-acetylglucosamine chains on as exemplified by the capacity of anti-HS mAb, IB4, MAL-1 and sWGA to bind the glycocalyx of these vessels whilst α-2,6-linked sialic acid residues (SNA ligand) could not be detected (Figures 1B,C). Interestingly, hyaluronan was minimally associated with lymphatic glycocalyx of naïve LVs (Figures 1B,C) but strongly expressed by interstitial cells morphologically resembling to macrophages or dendritic cells (Supplementary Figure 2). Of note, the deposition of those glycocalyx moieties was neither associated specific morphological structures of initial lymphatic ECs nor with the low expression regions of basement membrane of LVs (28). Further analysis of our images, however, demonstrated that the intensity of fluorescence of anti-HS Ab, IB4, and sWGA (but not MAL-1) was inversely proportional to the vessel size (Figure 1D); suggesting that HS, α-D-galactosyl and N-acetylglucosamine moieties were more abundant on small initial capillaries than in larger vessels, whilst sialic acid levels were constant. Interestingly, similar pattern of expression of HS, α-D-galactosyl moieties, α-2,3-linked sialic acid residues and N-acetylglucosamine chains, were observed on the LVs of the mouse ear skin (Figures 2A–C) but also on the LVs in human breast skin tissue sections (Figures 2D,E). When comparing the intensity of fluorescence of the lectins binding to initial lymphatics and blood vessels (post-capillary venules) of the mouse cremaster muscles, we observed that both vasculatures were characterized by the presence of α-D-galactosyl moieties, α2,3-linked (but not α2,6-linked) sialic acids and N-acetylglucosamine chains. Of note, we noticed that IB4 had a greater affinity for the glycocalyx of blood post-capillary venules whilst MAL-1 showed a trend toward a higher binding to LVs (Figures 3A,B).

Collectively, our data demonstrate that *in vivo* the glycocalyx of initial lymphatic capillaries includes HS, α-D-galactosyl moieties, sialic acid α-2,3-linked glycans and, to a lesser extent N-acetylglucosamine residues in both mouse and human tissues.

### The Glycocalyx of Initial Lymphatic Vessels Is Remodeled During Inflammation

**Figure 4:**
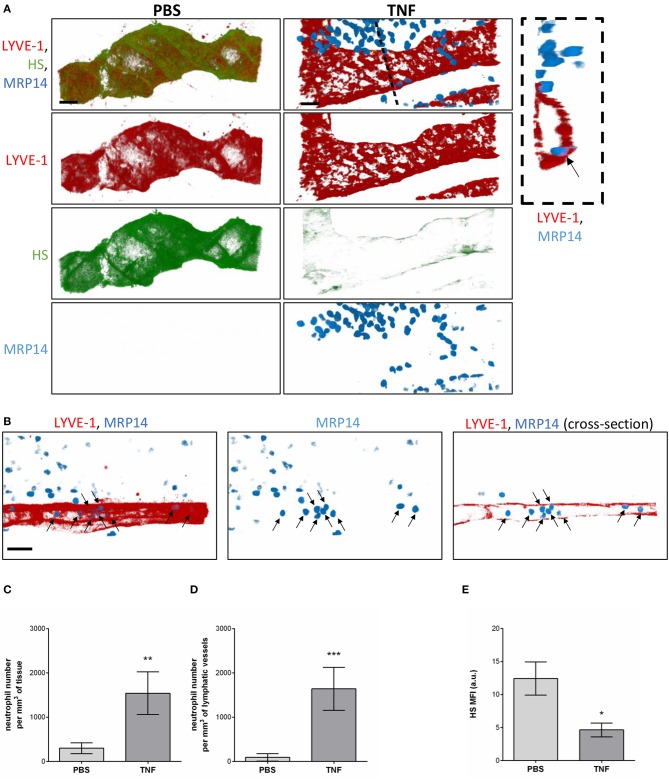
Regulation of Heparan Sulfate expression in the glycocalyx of initial lymphatics and neutrophil migration responses upon inflammation. WT mice were intrascrotally (i.s.) injected with TNF (300 ng) and the inflammatory response was allowed to develop for 16 h. Two hours before the end of the inflammation period, mice were further injected i.s with anti-HS (green) and anti-LYVE-1 (red) Abs to visualize the heparan sulfate and lymphatic vasculature, respectively. The cremaster muscles were then harvested, fixed, permeabilized, and immunostained with an anti-MRP14 mAb to visualize the neutrophils (blue). Unstimulated controls received an i.s injection of PBS. **(A)** Representative 3D-reconstructed confocal images of an initial lymphatic vessel from PBS- (left panels) or TNF- (right panels) stimulated tissues. The image on the far right is a transversal cross-section view of the TNF-stimulated tissue image along the dotted line and showing the entry of a neutrophil (arrow) within the lymphatic vessel. **(B)** Representative 3D-reconstructed confocal images of an initial lymphatic vessel from a TNF-stimulated tissues. The right hand side panel is a longitudinal cross-section of the lymphatic capillary demonstrating the presence of numerous neutrophils (arrows) within the lymphatic vessel. **(C)** Quantification of the number of neutrophils that have infiltrated the interstitial tissues (per mm^3^ of tissue). **(D)** Quantification of the number of neutrophils present within lymphatic vessels (per mm^3^ of lymphatic vessel). **(E)** Quantification of the Mean Fluorescence Intensity (MFI) of HS expression on lymphatic vessels following PBS and TNF stimulation. Results are from *n* = 8–12 vessels per mouse with 3–6 animals per group. Statistically significant differences between isotype control PBS and TNF-treated groups are indicated by asterisks: *P < 0.05, **P < 0.01, ***P < 0.001. Bars = 50 μm.

**Figure 5:**
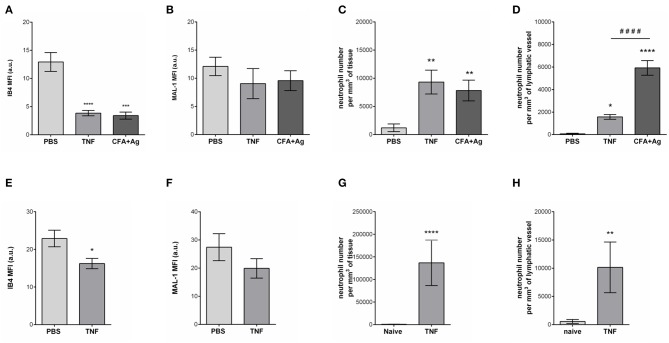
Selective cleavage of initial lymphatic vessels glycocalyx during acute inflammation. The cremaster muscles of mice were stimulated following intrascrotal (i.s.) injection of TNF (300 ng) or CFA + Ag (200 μg). Control mice were injected with PBS. For 2 h before the end of the inflammation period, mice were further injected i.s. with IB4 or MAL-1 and anti-LYVE-1 Abs to reveal glycan chains and lymphatic vessels, respectively. At the end of the inflammation period, the cremaster muscles were harvested, fixed, and immunostained for neutrophils (anti-MRP14 mAb) prior to the visualization and quantification of the inflammatory response by confocal microscopy. For ear stimulation, the dorsal skin of mouse ears were injected with TNF (300 ng); and 16 h later, ears were harvested, fixed and stained whole-mount with fluorescently-labeled IB4 or MAL-1; anti-LYVE-1 and anti-MRP14 mAbs to reveal glycan chains, lymphatic vessels and neutrophils, respectively, prior to the visualization and quantification of the inflammatory response by confocal microscopy. Control mice were injected with PBS. The mean fluorescence intensity (MFI) for IB4 or MAL-1 binding to the lymphatic glycocalyx was quantified by creating an isosurface on lymphatic vessel channel (LYVE-1), excluding the signal from MRP14 channel using IMARIS software. **(A)** Mean fluorescence intensity (MFI) for IB4 staining on cremaster lymphatic vessels. **(B)** Mean fluorescence intensity (MFI) of MAL-1 staining on cremaster lymphatic vessels. **(C)** Number of neutrophils into the cremaster interstitium. **(D)** Number of neutrophils into the cremaster initial lymphatic vessels. **(E)** Mean fluorescence intensity (MFI) for IB4 staining on ear skin lymphatic vessels. **(F)** Mean fluorescence intensity (MFI) of MAL-1 staining on ear skin lymphatic vessels. **(G)** Number of neutrophils in the ear skin interstitium. **(H)** Number of neutrophils within the ear skin initial lymphatic vessels. Results are from *n* = 4–6 vessels per tissue with at least 5 animals per group. Statistically significant differences between stimulated and unstimulated treatment groups are indicated by: *P < 0.05, **P < 0.01, ***P < 0.001, ****P < 0.0001. Statistically significant differences between TNF-stimulated and CFA + Ag-stimulated tissues are indicated by: ^####^*P* < 0.0001.

Having observed the binding of the anti-HS mAb, IB4, and MAL-1, but not SNA to the lymphatic capillaries *in vivo*, we next investigated the potential regulation of LV glycocalyx during acute inflammation. For this purpose, the cremaster muscles of mice were first subjected to acute TNF-induced inflammation, a cytokine we have previously shown to induce the rapid migration of neutrophils into the tissue-associated lymphatic vessels (26). For this purpose, the cytokine was injected i.s. for 16 h prior to fluorescent staining of tissues with anti-HS Ab, anti-LYVE-1 and anti-MRP-14 mAb to visualize HS, lymphatic vasculature and neutrophils, respectively. Our data showed that TNF-stimulation induces the rapid migration of neutrophils into the tissue and the lymphatic vasculature (Figures 4A–D). Of note, we did not observed preferred entry sites for neutrophils migration within LVs and with regards to glycocalyx components. However, we detected a significant decrease (~64%) in HS expression on LVs of TNF-stimulated tissues as observed by a reduced binding of the anti-HS mAb (Figures 4A,E). Similarly, we observed a reduction in staining for IB4 but not MAL-1, suggesting a decrease in galactosyl residues (~3-fold decrease as compared to unstimulated lymphatic glycocalyx) but not sialic acid α-2,3-linked glycans following TNF-stimulation (Figures 5A,B). Similar results were obtained in another inflammatory model as induced by antigen sensitization (i.e., injection of an emulsion of ovalbumin in Complete Freund's Adjuvant, CFA + Ag) (Figures 5A,B). This remodeling of LV glycocalyx was associated with the recruitment of neutrophils into the interstitium and the tissue-associated lymphatic vessels in both inflammatory models (Figures 5C,D). Finally, to confirm that the LV glycocalyx remodeling was not restricted to inflamed cremaster muscles, similar analyses were performed in the mouse ear dorsal skin (Figures 5E–H). Our data clearly demonstrate that ear skin LVs also exhibit a cleavage of α-D-galactosyl moieties but not sialic acids following TNF-stimulation; a response associated with neutrophil infiltration into the tissue and migration into LVs (Figures 5E–H).

Collectively, these results provide evidence for moiety-specific remodeling of the LV glycocalyx in two distinct vascular beds, a response that is associated with neutrophil trafficking into inflamed tissues and LVs.

### Neutrophils Do Not Contribute to the Remodeling of the LEC Glycocalyx of Initial Lymphatic Vessels

**Figure 6:**
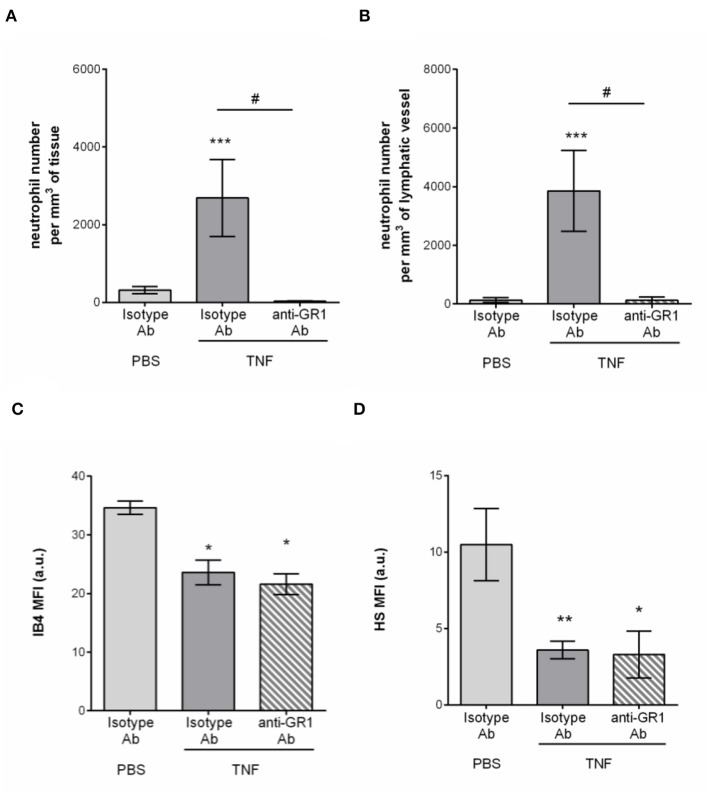
Neutrophil-independent remodeling of initial lymphatic glycocalyx upon TNF-induced inflammation. Circulating neutrophils were depleted via a daily intraperitoneal (i.p.) injection of the anti-GR1 depleting antibody (25 μg) for 3 days prior to the induction of the inflammatory response. Inflammation of the cremaster muscles of WT mice was induced by the intrascrotal (i.s.) injection of TNF (300 ng) for 16 h. Non-depleted control mice received i.p. injections of an isotype control antibody; and unstimulated animals were injected i.s. with PBS. Two hours before the end of the inflammation period, mice were i.s injected with fluorescently labeled IB4/anti-HS Ab in conjunction with a non-blocking anti-LYVE-1 Ab to visualize the glycocalyx moieties and the lymphatic vessels, respectively. At the end of the inflammatory period, mice were sacrificed and their cremaster muscles harvested, fixed, and fluorescently-labeled with an anti-MRP14 Ab to detect the neutrophils. Neutrophil migration responses were analyzed in 3D by confocal microscopy. Lymphatic glycocalyx mean fluorescence intensity (MFI) was quantified with an isosurface generated on the lymphatic vessels (LYVE-1^+^ vessels) using IMARIS software. **(A)** Number of neutrophils migrated into the tissue. **(B)** Number of neutrophils within the cremaster initial lymphatic vessels. **(C)** Mean fluorescence intensity (MFI) of IB4 staining on lymphatic vessels. **(D)** Mean fluorescence intensity (MFI) of anti-HS Ab staining on lymphatic vessels. Results are from at least 3 animals per group. Statistically significant differences between stimulated and unstimulated treatment groups are indicated by: *P < 0.05, **P < 0.05, ***P < 0.001. Statistically significant differences between neutrophil-depleted and non-depleted groups are indicated by: ^#^*P* < 0.01.

Having associated the remodeling of the glycocalyx of tissue-associated initial lymphatic capillaries with extensive neutrophil recruitment during acute inflammation, we then investigated the contribution of these leukocytes to this remodeling. Neutrophils from the blood circulation are known to secrete proteases (e.g., elastase) (29) and release reactive oxygen species (ROS) (30), that can cleave or modify glycoproteins at the surface of BECs during their recruitment (i.e., ICAM-1), thus contributing to the modification of the composition of the blood vascular glycocalyx. To directly assess a similar role of neutrophils during their entry into LVs, we performed antibody-based depletion of neutrophils prior to the induction of TNF-induced inflammation of the mouse cremaster muscles and analysis of the response (i.e., HS and IB4 expression profile, and neutrophil migration) by confocal microscopy. Antibody-based depletion efficiency was first confirmed by the absence of detection of neutrophils in the blood circulation (Supplementary Figure 3) and in the inflamed tissues and their associated LVs as compared to animals treated with an isotype-matched control Ab (Figures 6A,B). Interestingly, however, neutrophil-depleted animals showed a similar level of LV glycocalyx remodeling (reduced detection of both for α-D-galactosyl residues and HS) following TNF-stimulation that of non-depleted animals (Figures 6C,D). These observations suggest that neutrophils are not responsible for the shedding of HS and Galactosyl moieties present lymphatic glycocalyx during TNF-induced inflammation *in vivo*.

### Endogenous Heparanase Contributes to the Remodeling of Initial Lymphatic Glycocalyx

**Figure 7:**
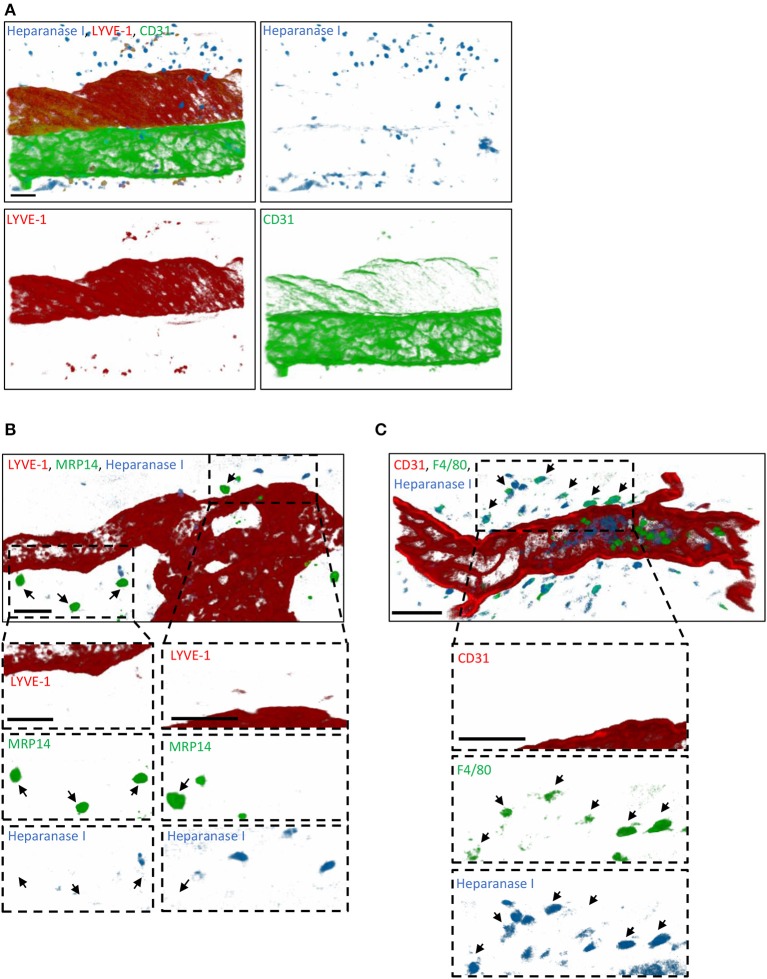
Cellular source of Heparanase I in inflamed cremaster muscles. Mouse cremaster muscles were stimulated with TNF (i.s. 300 ng) followed by whole-mount immunostaining of the tissues with fluorescently labeled antibodies to reveal the HS-degrading enzyme Heparanase I, the lymphatic and/or blood vasculatures, and neutrophils/macrophages prior to the visualization of the samples by confocal microscopy. **(A)** Representative 3D-reconstructed confocal images of a region of the cremaster muscle showing that Heparanase I (blue) is not associated with cremaster lymphatic (LYVE-1, red) or blood (CD31, green) vasculatures but with cells present within the interstitial tissue. **(B)** Representative 3D-reconstructed confocal images of a region of the cremaster immunostained for lymphatic vessels (LYVE-1, red), neutrophils (MRP14, green) and Heparanase I (blue). Two magnified regions (dotted box) within the main image are provided in the bottom panels demonstrating that Heparanase I is neither associated with lymphatic endothelial cells nor neutrophils (arrows). **(C)** Representative 3D-reconstructed confocal images of a region of the cremaster immunostained for endothelial cells (CD31, red), macrophages (F4/80, green), and Heparanase I (blue). A magnified region (dotted box) within the main image are provided in the bottom panels demonstrating that Heparanase I is strongly associated with macrophages (arrows). Bar = 40 μm. Images are representative pictures from at least 5 vessels/animals, with at least 4 animals.

Since tissue-infiltrated neutrophils did not contribute to the remodeling of lymphatic glycocalyx *in vivo*, we next sought to investigate the role of endogenous glycosidases in glycocalyx degradation. Interestingly, a study by Schmidt et al. has demonstrated that the HS-specific endoglycosidase, Heparanase, is responsible for the cleavage of the glycan layer on the luminal side of blood capillaries in the lung during sepsis (16). To address the hypothesis that this enzyme may also be involved in the remodeling of the initial lymphatic glycocalyx during an acute inflammatory response, we first investigated its cellular source. For this purpose, TNF-stimulated cremaster muscles were immunostained with antibodies against Heparanase I (or with an isotype control antibody, Supplementary Figure 4), lymphatic (LYVE-1) and blood (CD31^high^) vasculatures and neutrophils (MRP14) or macrophages (F4/80) prior to analysis by confocal microscopy. Heparanase I is an endo-β-glucuronidase implicated in the degradation of HS chains and known to be expressed by leukocytes, platelets and blood endothelial cells (31–33). In our *in vivo* inflammatory model, confocal image analyses showed that this enzyme was not associated with LVs post-TNF-stimulation but with interstitial cells (Figure 7A). In fact, Heparanase I was strongly detected in macrophages whilst the tissue-infiltrated neutrophils did not exhibit a positive immunostaining for this enzyme (Figures 7B,C). Similar pattern of Heparanase I expression was observed at an early (8 h) time-point of the inflammatory reaction (Data not shown).

**Figure 8:**
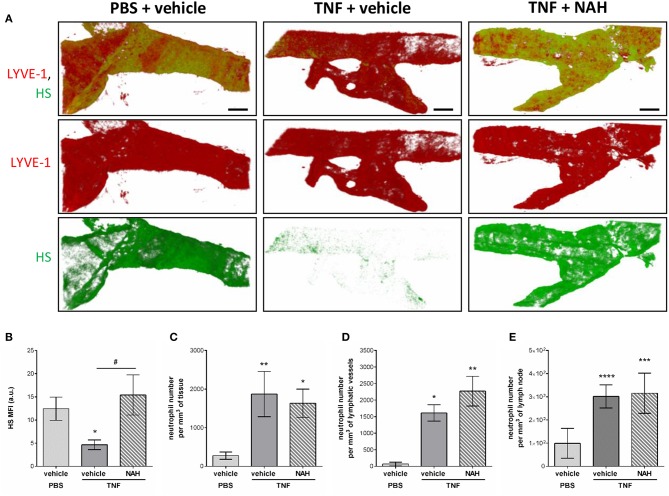
Effect of a non-anticoagulant heparanase inhibitor (NAH) on TNF-induced HS remodeling of the lymphatic glycocalyx and neutrophil migration responses. TNF (300 ng) or PBS (as control) were administrated intrascrotally (i.s.). Three hours later, mice received an i.s. injection of 50 μg of non-anticoagulant heparanase inhibitor N-desulfated/re-N-acetylated heparin (NAH) or vehicle. At 14 h post-TNF-stimulation, mice were further i.s injected with anti-HS (green) and anti-LYVE-1 (red) mAbs to label *in vivo* and visualize the heparan sulfate layer and lymphatic vasculature, respectively. Sixteen hours post-TNF-stimulation, animals were sacrificed, their cremaster tissues and draining (i.e. inguinal) lymph nodes harvested and prepared for confocal microscopy to measure HS remodeling on tissue-associated initial lymphatic vessels and neutrophil migration responses. **(A)** The images show representative 3D-reconstructed confocal images of an initial lymphatic vessel from a PBS (left hand side panels) or TNF (middle and right-hand side panels)-stimulated tissues with or without NAH-pre-treatment (*N* = 3–6 mice per group; Bars = 50 μm). **(B)** Quantification of the Mean Fluorescence Intensity (MFI) of HS expression on initial lymphatic vessels of the cremaster muscles (*N* = 3–6 mice per group). **(C)** Quantification of the number of neutrophils present into the tissue's interstitium (per mm^3^ of tissue) (*N* = 6–14 mice per group). **(D)** Quantification of the number of neutrophils present within initial lymphatic vessels (per mm^3^ of lymphatic vessel) (*N* = 5–10 mice per group). **(E)** Quantification of the number of neutrophils present in the draining lymph nodes of the cremaster muscles (per mm^3^ of lymph node) (*N* = 3 mice per group with two dLNs per animals). Statistical significance between PBS and TNF stimulated groups are indicated by asterisks: *P < 0.05, **P < 0.01, ***P < 0.001, ****P < 0.0001. Statistical significance between vehicle and NAH-treated groups are indicated by hash symbol: ^#^*P* < 0.05.

To get further mechanistic insights into the role of Heparanase into the remodeling LV HS during inflammation, we tested the effect of local injection of a non-anticoagulant heparanase inhibitor N-desulfated/re-N-acetylated heparin (NAH). Briefly, TNF-stimulated cremaster muscles were treated locally (i.s. injection) with NAH, or its vehicle, 3 h post-administration of TNF. Two hours before the end of the inflammatory period (i.e., 16 h), the tissues were fluorescently immunostained with anti-LYVE-1 and anti-HS Abs to visualize the lymphatic vasculature and HS, respectively. In addition, at the end of the *in vivo* test period, tissues were harvested, fixed and immunostained with an anti-MRP14 Ab to enable quantification of neutrophil migration responses. In line with our previous results (Figure 4), vehicle-treated tissues exhibited a decrease in anti-HS Ab immunostaining on initial LVs upon TNF-stimulation (Figures 8A,B). Interestingly, inflamed tissues treated with NAH showed a similar deposition of HS as found in un-stimulated cremaster muscles (Figures 8A,B), confirming the role of endogenous heparanase for the remodeling of LEC glycocalyx during TNF-induced inflammation *in vivo*. In contrast, however, local administration of NAH had no significant impact on neutrophil migration into tissues and tissue-associated LVs as compared to vehicle-treated animals (Figures 8C,D). Furthermore, NAH-treatment did not inhibit neutrophil trafficking to the cremaster draining lymph nodes (Figure 8E). Collectively, these results suggest that local inhibition of heparanase-dependent shedding of HS on initial LVs does not affect the capacity of neutrophils to infiltrate the tissue-associated lymphatic vasculature.

### Blockade of Initial LV Glycocalyx Shedding Prevents Local Fluid Drainage of TNF-Stimulated Cremaster Muscles

**Figure 9:**
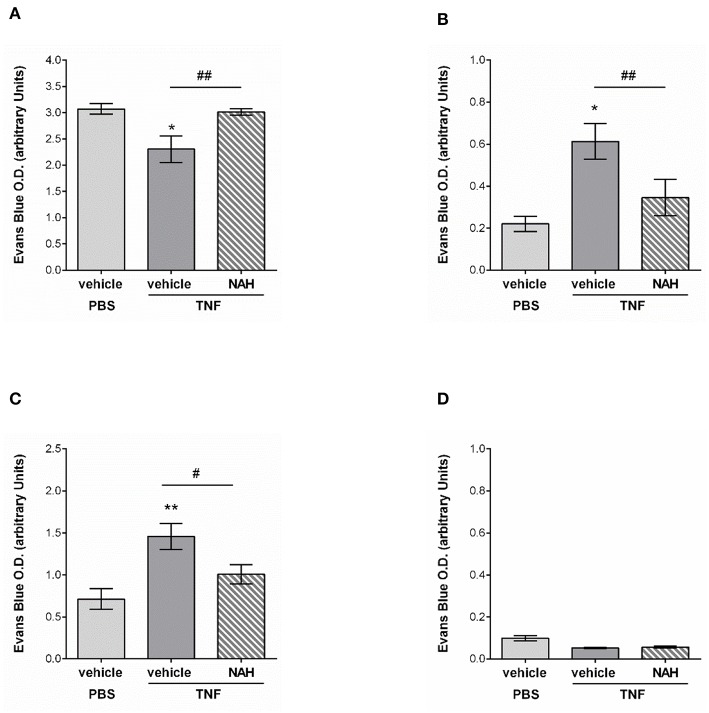
Effect of a non-anticoagulant heparanase inhibitor (NAH) on lymphatic drainage in TNF-stimulated tissues. TNF (300 ng) or PBS (as control) were administrated intrascrotally. Three hours later, mice received an i.s. injection of 50 μg of non-anticoagulant heparanase inhibitor N-desulfated/re-N-acetylated heparin (NAH) or vehicle. Twenty min before the end of the inflammatory period (i.e., 16 h), mice received an i.s. injection of 1% Evans Blue. Animals were then sacrificed, their plasma, cremaster tissues, draining, and non-draining lymph nodes were collected and prepared for spectrophotometric analysis of Evans Blue content. **(A)** Quantification of the Evans Blue content in the mouse cremaster (*N* = 6–10 mice per group). **(B)** Quantification of the Evans Blue content in draining lymph (i.e., inguinal) nodes of the cremaster muscles (*N* = 6–7 mice per group). **(C)** Quantification of the Evans Blue content in mouse plasma samples (*N* = 6–10 mice per group). **(D)** Quantification of the Evans Blue content in non-draining (i.e., brachial) lymph nodes (*N* = 4 mice per group). Statistical significance between PBS and TNF stimulated groups are indicated by asterisks: *P < 0.05, **P < 0.01. Statistical significance between vehicle and NAH-treated groups are indicated by hash symbol: ^#^*P* < 0.05, ^##^*P* <0.01.

Whilst innate immune cell trafficking is an important aspect of lymphatic biology, a key function of tissue-associated initial lymphatic capillaries is to transport fluids and macromolecules (including antigens) out of the interstitium into dLNs (for immune surveillance) and back into the blood circulation. Tissue drainage by LVs naturally occurs at steady state but also during inflammatory responses; but can be impaired during pathological conditions, such as aging (20). Nevertheless, the current literature lacks convincing *in vivo* evidence for a role of lymphatic glycocalyx in this phenomenon in the context of acute inflammation. The aim of this last set of experiments was thus to determine how the remodeling of the lymphatic glycocalyx influences the draining capabilities of these vessels. For this purpose, we investigated the draining function of initial lymphatic capillaries of mouse cremaster muscles by injecting locally (i.s.) an isotonic solution of Evans Blue (EB) before measuring the quantity of the dye in cremaster muscles, draining and non-draining LNs as well as in the plasma of animals treated locally with the heparanase inhibitor NAH. Lymphatic drainage of EB was first confirmed by direct visualization of the dye in the lymph nodes and lymphatic venules (i.e., inguinal) draining the cremaster muscles (Supplementary Figure 5A). These observations were supported by the significant reduction of EB levels (~30%) in TNF-stimulated cremaster muscles as compared to non-inflamed tissues (Figure 9A). Interestingly, whilst in non-inflamed conditions NAH did not modify EB drainage (Supplementary Figure 5B), treatment of TNF-stimulated tissues with this inhibitor restored EB levels to that seen in non-inflamed control tissues (Figure 9A). Furthermore, whilst TNF stimulation led to an increase in EB detection in dLNs (~180% increase) and plasma (~100% increase), as compared to unstimulated tissues, these responses were significantly suppressed in NAH-treated mice (Figures 9B,C). Of note, no EB could be detected in non-draining (i.e., brachial) LNs (Figure 9D), suggesting that our responses is mainly due to lymphatic drainage rather than potential diffusion of the locally-injected dye into blood capillaries. Furthermore, to exclude the possibility that blood vascular hyper-permeability may increase interstitial pressure (and thus, lymphatic drainage) within the tissues during inflammation, we sought to investigate the extent of blood vascular leakage at time of glycocalyx remodeling and lymphatic drainage using the Miles assay. Briefly, mice were injected with TNF (or PBS as control) prior to be treated locally with the heparanase inhibitor NAH (or vehicle control) 3 h later. Two hours before the end of the inflammatory period (i.e., 16 h), animals received an intravenous injection of 0.5% of Evans blue. The quantity of dye present in the cremaster muscles was then assessed by spectrophotometry. Our data show that of the accumulation of Evans blue in tissues was similar between unstimulated and TNF-stimulated groups (Supplementary Figure 6). Furthermore, NAH treatment did not affect vascular permeability response at this time point. These observations are supported by a previous study from our group demonstrating that *in vivo*, TNF promote a rapid but transient blood vascular leakage during the first 30 min post-stimulation (27). Together, these results suggest that the enhanced lymphatic drainage response that we observed during glycocalyx remodeling upon TNF-inflammation occurred independently of blood vascular leakage at the time-point analyzed.

Collectively, these results suggest that protecting the lymphatic glycocalyx HS from heparanase-induced shedding during TNF-induced inflammation reduces the capacity of initial lymphatic vessels to drain interstitial fluids toward draining lymph nodes and back into the blood circulation.

## Discussion

The lymphatic vasculature is the second circulatory system of high vertebrates and is formed by a unidirectional network of vessels and dLNs that starts in tissues with blunt-ended initial lymphatic capillaries. The main function of these specialized vessels is to remove interstitial fluids and macromolecules to counteract tissue edema and as such are essential for tissue homeostasis (19). Initial lymphatic capillaries also play a key role in immune surveillance by allowing not only the rapid drainage of antigens and migration of professional antigen-presenting cells, such as DCs and macrophages but also neutrophils toward the dLNs in order to initiate adaptive immune responses (26, 34). Initial lymphatic capillaries are composed of a monolayer of endothelial cells (LECs) that share some molecular similarities with blood endothelial cells (BECs) but also a few architectural and phenotypic differences (19). Furthermore, whilst both BECs and LECs contain a carbohydrate-rich glycocalyx layer on their cell surface, the characteristics of the glycocalyx of initial lymphatic vessels *in vivo* and its role in acute inflammation is unknown.

In an effort to gain insight into the characteristics of the composition of glycan residues forming the glycocalyx of initial lymphatic vessels, an antibody targeting the most abundant glycan moiety of endothelial glycocalyx, HS, alongside a panel of 4 lectins (IB4, MAL-1, SNA and sWGA) were used *in vivo* in the mouse cremaster muscle. Lectins are the most commonly employed glycan-binding glycoproteins used to label the blood vascular glycocalyx, especially IB4 that binds α-D-galactosyl moieties present at the luminal surface of microvascular endothelial cells (35). In contrast, lectins have not been systematically used to investigate the LEC glycocalyx *in vivo*. In our study, we demonstrate that IB4, MAL-1 and to a lesser extent sWGA, can successfully bind lymphatic vessels. Similar pattern of expression of those glycocalyx moieties was observed to be also present in lymphatic vessels from the mouse murine ear skin and in human breast skin samples. Interestingly, we noted a higher staining for IB4 on blood vessels than on initial lymphatic capillaries of mouse cremaster muscles. This difference in the binding level of IB4 on these two distinct vasculatures could be attributed to the fact that blood vessel walls are surrounded by smooth muscle cells and pericytes known to exhibit IB4-binding carbohydrate residues in their own glycocalyx (35) that would be also revealed by the local delivery of the lectin employed in our study. In contrast, initial lymphatic vessels are usually devoid of perivascular cells and are only composed of a monolayer of LECs surrounded by a thin and perforated basement membrane (28), hence the overall lower detection of α-D-galactosyl moieties on initial lymphatics as compared to blood vessels. In contrast, MAL-1 but not SNA, both of which have been used in a plethora of studies to detect different sialic acid-linked glycans (36–39) was found to bind the LEC glycocalyx to the same extent as BEC glycocalyx. MAL-1 expression on lymphatic glycocalyx could be related to the expression of lymphatic glycoproteins, such as of LYVE-1 and podoplanin by LECs, molecules exhibiting high levels of α2,3-linked sialic acid moieties (40, 41). In support of our findings, a study by Nightingale et al. showed that the level of MAL-1 binding to the surface of human dermal LECs *in vitro* was higher than of SNA (40), whilst being similar for other cell types (42). Altogether, these results suggest that the glycocalyx of LECs exhibit more α2,3-sialic acid linked moieties (i.e., MAL-1 ligands) than α2,6-sialic acid linked glycans (i.e., SNA ligand).

We further characterized the glycocalyx of tissue lymphatic capillaries by revealing the presence of HS on those vessels. HS proteoglycans represent the majority of all proteoglycans expressed by BECs, and as such HS is the major constituent of the blood vascular glycocalyx, representing more than 50% of the vascular GAGs present on those vessels (8). GAGs have the capacity to bind and immobilize chemokines for leukocyte recruitment to blood vessels. This is particularly the case for CXCL1 and CXCL2, two potent neutrophil chemoattractants (11, 43). HS was also shown to protect endothelial cells from oxidative stress damage by quenching reactive oxygen species (ROS) through the binding of superoxide dismutase enzyme to GAG HS and to maintain nitric oxide bioactivity (44). In the lymphatic system, HS has been implicated in the formation of a CCL21 gradient within the interstitium in the vicinity of the LVs in order to direct dermal DCs toward LVs, as indirectly demonstrated by the inhibitory effect of a bacterial heparinase on CCL21 gradient formation and DC recruitment to LVs (45). Similarly, transgenic mice exhibiting impairment in HS synthesis showed a defect in DC and naïve T-cell trafficking into the lymph nodes via high endothelial venules (13). In this context, we have recently demonstrated that during TNF-induced inflammation and antigen challenge, tissue-infiltrated neutrophils rapidly migrate into the lymphatic system through initial lymphatic capillaries (26), a process occurring in a strictly CCL21/CCR7 dependent manner. However, at present little is known about the regulation and role of tissue-associated lymphatic glycocalyx and HS during inflammation, and it is still unclear whether the LEC glycocalyx can regulate the migratory response of these leukocytes, a topic that needs further explorations.

Another important GAG associated with lymphatic vessels is Hyaluronan (HA). In fact, LECs from initial lymphatics are characterized by the expression of the specific marker LYVE-1, a transmembrane molecule that is known to be the lymphatic receptor for HA (23). Recently, an elegant study by Johnson et al. showed that LYVE-1 serves as a docking structure for migrating DCs. Specifically, the authors propose that HA, secreted by DCs, create a bridge between the receptor LYVE-1 and CD44 on LECs and the leukocytes, respectively (46). Whilst HA and CD44 are important for neutrophil adhesion to blood vessels (47, 48), to date, there is no evidence of a similar mechanism for neutrophil interaction with LECs. In fact, most studies have demonstrated that neutrophils preferentially use β2-integrins (binding to ICAM-1 on LECs) during their migration into lymphatic vessels (26, 49, 50).

There is an abundance of literature reporting the remodeling of the blood vascular glycocalyx during inflammation. Specifically, the BEC glycocalyx and in particular HS proteoglycans are rapidly shed in response to inflammation as induced by cytokines, such as TNF, but also in various experimental and pathological inflammatory conditions, such as ischemia reperfusion injury or sepsis (8, 51, 52). HS degradation during inflammation appears to allow the exposure of underlying adhesion molecules (e.g., ICAM-1, VCAM-1) and the release of pro-inflammatory chemokines, thus facilitating neutrophil adhesion and extravasation through the blood vessel wall (4, 16). Conversely, an intact layer of HS proteoglycans in physiological and homeostatic conditions has been associated with inhibition of neutrophil adhesion to BECs (16). Mechanistically, cleavage of BEC glycocalyx has been suggested to be partially as a consequence of the activation of leukocytes, such as neutrophils through their release of enzymes and ROS (15, 30, 53). However, there is no evidence to date of a similar phenomenon during acute inflammation at the level of tissue-associated lymphatic capillaries. Of relevance however, Zolla et al. have recently described the thinning of the glycocalyx of mesenteric afferent lymphatic collecting venules in aged rats (20). Here, we provide the first conclusive evidence for reduced HS and α-D-galacosyl moieties on initial lymphatic glycocalyx during acute inflammatory responses elicited in the mouse cremaster muscle. Furthermore, this response, elicited by TNF or antigen sensitization, was associated with the migration of neutrophils into the lymphatic vasculature. Interestingly, in neutrophil-depleted animals, the degradation of the lymphatic glycan layer still occurred, suggesting that in contrast to the cleavage of the BEC glycocalyx, this phenomenon in initial lymphatic capillaries is not mediated by neutrophils. In exploring other mechanistic pathways, we investigated the cellular source of Heparanase I, a β-D-endoglucuronidase known to be the enzyme capable to cleave HS in mammalian systems (31–33). Heparanase expression is mainly restricted to platelets and activated leukocytes, such as T-cells, macrophages, DCs and neutrophils (33), but can also be upregulated in other cell types during chronic inflammatory disorders, including BECs (54). Furthermore, neutrophil Heparanase expression, has been associated with degradation of sub-endothelial extracellular matrix of blood vessels (55, 56). However, in our acute inflammatory model, Heparanase I was neither associated with LECs nor with extravasated neutrophils but was found to be highly expressed by interstitial macrophages. Altogether, these set of data could suggest a distinct function of this enzyme during the interaction of this leukocyte with blood vessels vs. lymphatic vessels. This hypothesis is supported by our novel findings using neutrophil-depletion experiments and showing that these leukocytes are not responsible for HS remodeling of LVs during acute inflammation *in vivo*. In deciphering further the mechanisms of HS degradation, we demonstrated that a specific non-anticoagulant inhibitor of the endoglycosidase heparanase (NAH) blocked the shedding of HS GAGs on initial LVs. These results are supported by a study from Schmidt et al. who showed that endogenous TNF catalyzed the degradation of HS constituents of the BEC glycocalyx in a heparanase-dependent manner in an experimental model of sepsis-induced acute lung injury (16). Similarly, Lukasz et al. demonstrated both *in vitro* and *in vivo* that angiopoietin-2 can induce the secretion of heparanase by BECs, thus contributing to the cleavage of BEC glycocalyx and resulting in an increase in blood vascular leakage and leukocyte diapedesis (57). In our study however, inhibition of glycocalyx cleavage did not interfere with the capacity of neutrophils to migrate into tissue-associated LVs, offering a prominent difference in the functional roles of lymphatic vs. blood vessel glycocalyx for the trafficking of these leukocytes. Since HS plays an important role in the migration of DCs (45), it is also possible that our findings suggest potential differences in the function of HS with respect to controlling the recruitment of innate immune cells to LVs as compared to neutrophils.

To further understand the physiological relevance of glycocalyx shedding for lymphatic function during inflammation, we analyzed the capacity of initial LVs to drain interstitial fluids and macromolecules out of inflamed tissues. Here, we showed that glycocalyx remodeling on initial lymphatic capillaries was directly associated with a rapid decrease in Evans Blue (EB) dye (locally injected to mimic tissue edema) detection in inflamed cremaster muscles. More importantly the reduced level of EB in those tissues was inversely associated with an increase in EB detection in cremaster draining lymph nodes and within the blood circulation. Of note we did not observed differences in blood vascular permeability at this time point (i.e., 16 h post-TNF stimulation) between stimulated and unstimulated (or with NAH-treatment); suggesting that the enhanced lymphatic drainage was not the consequence of an increase in interstitial pressure due to vascular permeability but related to glycocalyx remodeling. This results is supported by our previous publication demonstrating that TNF-induced blood vascular leakage is a rapid but transient phenomenon occurring within the first 30 min post-cytokine stimulation (27). Interestingly, however, in TNF-stimulated tissues local blockade of HS shedding with the heparanase inhibitor, NAH, led to the detection of high levels of EB in the cremaster muscles, and conversely, a lower quantity in draining lymph nodes and plasma of the treated animals. Supported by the fact that EB is highly negatively charged, these results suggest that the glycocalyx of initial lymphatic capillaries may form an electrostatic barrier to interstitial solutes and macromolecules similarly to blood vessels due to the presence of negative electric charges of GAG molecules present on LEC glycocalyx (8, 58). The degradation of the lymphatic glycocalyx could therefore contribute to the rapid drainage of excessive interstitial fluids generated by the inflammatory response, and to promote the rapid transport of antigens toward the closest draining lymph node in order to mount an adaptive immune response as required. in sharp contrast, during aging, the thinning of the glycocalyx of large lymphatic collectors and the loss of extracellular matrix in the valve area of these vessels (20) were associated with a decrease in the capacity of lymphatic collectors to transport fluids correctly due to reduced investiture of smooth muscle cells and pericytes around the valves (59). However, our study clearly shows that the cleavage of initial lymphatic glycocalyx is important for more efficient drainage of the tissues. Initial lymphatic capillaries are in fact structurally distinct from lymphatic collectors (60, 61). Specifically, they exhibit an oak leaf-shaped monolayer of overlapping endothelial cells facilitating the rapid absorption of fluids and macromolecules as well as contributing to the migration of immune cells (19). Initial lymphatics are also mostly devoid of perivascular cells in contrast to the large collector vessels that are covered with pericytes and smooth muscle cells. Initial lymphatics also express specific and highly glycosylated molecules, such as LYVE-1 (usually absent or reduced on collecting LVs) (22). Taken together, the unique molecular and architectural characteristics of initial lymphatics may suggest differences in the composition of the glycocalyx that may exhibit different functions as compared to the glycocalyx of large collecting venules. This is categorically supported by our findings that the cleavage of HS GAGs on initial lymphatic capillaries promotes a faster removal of interstitial fluids from inflamed tissues.

In conclusion, our study has revealed the presence of α-D-Galactosyl moieties, α2,3-sialic acid-linked glycans and HS as key components of the initial lymphatic capillary glycocalyx *in vivo*. We also demonstrate for the first time that HS and α-D-Galactosyl moieties are cleaved from the LEC glycocalyx upon inflammation, a response that appears to be mediated by endogenous heparanase activity. Interestingly, the shedding of glycocalyx components was not associated with enhanced neutrophil interaction and recruitment to lymphatic vessels, a response that is in sharp contrast with the importance of BEC glycocalyx cleavage for the migration of leukocytes through blood vessels (4). However, our data suggest that inflammation-induced shedding of initial lymphatic glycocalyx is important for the rapid drainage of interstitial fluids and macromolecules out of inflamed tissues into the draining lymph nodes and back into the blood circulation. This response is essential for immune surveillance and for the development of a specific adaptive immunity against foreign soluble antigens. Conversely, this phenomenon may also help with the dissemination of small pathogens and pro-inflammatory mediators into the body, thus potentially contributing to the induction of a rapid systemic inflammatory response.

## Data Availability Statement

The datasets generated for this study are available on request to the corresponding author.

## Ethics Statement

All animal experiments were approved by the local biological service unit Ethical Committee at Queen Mary University of London and carried out under the Home Office Project Licenses (70/7884 165 & P873F4263) according to the guidelines of the United Kingdom Animals Scientific Procedures Act (1986).

This study was carried out in accordance with the recommendations of the NHS Health Research Authority and the NRES Committee East of England–Cambridge Central committee (15/EE/0192) with written informed consent from all subjects. All subjects gave written informed consent in accordance with the Declaration of Helsinki. The protocol was approved by the NRES Committee East of England–Cambridge Central.

## Author Contributions

M-BV provided the overall project supervision by designing and performing experiments, analyzing the data, and writing the manuscript. SA performed the experiments, analyzed the data, and contributed to the writing of the manuscript. RK, HB, and RP performed some experiments. WW secured the funding for SA and contributed to the supervision of the project. SN provided the valuable tools, secured funding for SA, and contributed to the supervision of the project and writing of the manuscript.

### Conflict of Interest

The authors declare that the research was conducted in the absence of any commercial or financial relationships that could be construed as a potential conflict of interest.

## Abbreviations

BEC: blood endothelial cell
CS: chondroitin sulfate
CFA: complete Freund's adjuvant
dLN: draining lymph node
GAG: glycosaminoglycan
HS: heparan sulfate
IB4: isolectin-B4
ICAM-1: intercellular adhesion molecule 1
i. s.: intrascrotal
LEC: lymphatic endothelial cell
LV: lymphatic vessel
PSGL-1: P-selectin glycoprotein ligand-1
MRP14: myeloid related protein 14
NAH: non-anticoagulant heparanase inhibitor N-desulfated/re-N-acetylated heparin
ROS: reactive oxygen species
TNF: tumor necrosis factor (alpha)
MAL-1: *Maackia amurensis* Lectin-1
SNA: *Sambucus nigra* agglutinin
sWGA: succinylated wheat germ agglutinin
VCAM-1: vascular cell adhesion molecule 1.

## References

[1] Vink H Duling BR. Identification of distinct luminal domains for macromolecules, erythrocytes, and leukocytes within mammalian capillaries. Circ Res. (1996) 79:581–9. 10.1161/01.RES.79.3.581 8781491

[2] Van Haaren PM Vanbavel E Vink H Spaan JA. Localization of the permeability barrier to solutes in isolated arteries by confocal microscopy. Am J Physiol Heart Circ Physiol. (2003) 285:H2848–56. 10.1152/ajpheart.00117.2003 12907418

[3] Megens RT Reitsma S Schiffers PH Hilgers RH De Mey JG Slaaf DW. Two-photon microscopy of vital murine elastic and muscular arteries. Combined structural and functional imaging with subcellular resolution. J Vasc Res. (2007) 44:87–98. 10.1159/000098259 17192719

[4] Reitsma S Slaaf DW Vink H Van Zandvoort MA Oude Egbrink MG. The endothelial glycocalyx: composition, functions, and visualization. Pflugers Arch. (2007) 454:345–59. 10.1007/s00424-007-0212-8 17256154 PMC1915585

[5] Oohira A Wight TN Bornstein P. Sulfated proteoglycans synthesized by vascular endothelial cells in culture. J Biol Chem. (1983) 258:2014–21. 6337150

[6] Tarbell JM Weinbaum S Kamm RD. Cellular fluid mechanics and mechanotransduction. Ann Biomed Eng. (2005) 33:1719–23. 10.1007/s10439-005-8775-z 16389519

[7] Wang W. Change in properties of the glycocalyx affects the shear rate and stress distribution on endothelial cells. J Biomech Eng. (2007) 129:324–9. 10.1115/1.2720909 17536899

[8] Kolarova H Ambruzova B Svihalkova Sindlerova L Klinke A Kubala L. Modulation of endothelial glycocalyx structure under inflammatory conditions. Mediators Inflamm. (2014) 2014:694312. 10.1155/2014/694312 24803742 PMC3997148

[9] Van Den Berg BM Vink H Spaan JA. The endothelial glycocalyx protects against myocardial edema. Circ Res. (2003) 92:592–4. 10.1161/01.RES.0000065917.53950.75 12637366

[10] Chelazzi C Villa G Mancinelli P De Gaudio AR Adembri C. Glycocalyx and sepsis-induced alterations in vascular permeability. Crit Care. (2015) 19:26. 10.1186/s13054-015-0741-z 25887223 PMC4308932

[11] Wang L Fuster M Sriramarao P Esko JD. Endothelial heparan sulfate deficiency impairs L-selectin- and chemokine-mediated neutrophil trafficking during inflammatory responses. Nat Immunol. (2005) 6:902–10. 10.1038/ni1233 16056228

[12] Proudfoot AE. The biological relevance of chemokine-proteoglycan interactions. Biochem Soc Trans. (2006) 34:422–6. 10.1042/BST0340422 16709177

[13] Bao X Moseman EA Saito H Petryniak B Thiriot A Hatakeyama S. Endothelial heparan sulfate controls chemokine presentation in recruitment of lymphocytes and dendritic cells to lymph nodes. Immunity. (2010) 33:817–29. 10.1016/j.immuni.2010.10.018 21093315 PMC2996097

[14] Sundd P Pospieszalska MK Cheung LS Konstantopoulos K Ley K. Biomechanics of leukocyte rolling. Biorheology. (2011) 48:1–35. 10.3233/BIR-2011-0579 21515934 PMC3103268

[15] Henry CB Duling BR. TNF-α increases entry of macromolecules into luminal endothelial cell glycocalyx. Am J Physiol Heart Circ Physiol. (2000) 279:H2815–23. 10.1152/ajpheart.2000.279.6.H2815 11087236

[16] Schmidt EP Yang Y Janssen WJ Gandjeva A Perez MJ Barthel L. The pulmonary endothelial glycocalyx regulates neutrophil adhesion and lung injury during experimental sepsis. Nat Med. (2012) 18:1217–23. 10.1038/nm.2843 22820644 PMC3723751

[17] Wiesinger A Peters W Chappell D Kentrup D Reuter S Pavenstadt H. Nanomechanics of the endothelial glycocalyx in experimental sepsis. PLoS ONE. (2013) 8:e80905. 10.1371/journal.pone.0080905 24278345 PMC3835794

[18] Marki A Esko JD Pries AR Ley K. Role of the endothelial surface layer in neutrophil recruitment. J Leukoc Biol. (2015) 98:503–15. 10.1189/jlb.3MR0115-011R 25979432 PMC4569049

[19] Baluk P Fuxe J Hashizume H Romano T Lashnits E Butz S. Functionally specialized junctions between endothelial cells of lymphatic vessels. J Exp Med. (2007) 204:2349–62. 10.1084/jem.20062596 17846148 PMC2118470

[20] Zolla V Nizamutdinova IT Scharf B Clement CC Maejima D Akl T. Aging-related anatomical and biochemical changes in lymphatic collectors impair lymph transport, fluid homeostasis, and pathogen clearance. Aging Cell. (2015) 14:582–94. 10.1111/acel.12330 25982749 PMC4531072

[21] Levick JR Michel CC. Microvascular fluid exchange and the revised Starling principle. Cardiovasc Res. (2010) 87:198–210. 10.1093/cvr/cvq062 20200043

[22] Banerji S Ni J Wang SX Clasper S Su J Tammi R. LYVE-1, a new homologue of the CD44 glycoprotein, is a lymph-specific receptor for hyaluronan. J Cell Biol. (1999) 144:789–801. 10.1083/jcb.144.4.789 10037799 PMC2132933

[23] Jackson DG. Hyaluronan in the lymphatics: the key role of the hyaluronan receptor LYVE-1 in leucocyte trafficking. Matrix Biol. (2018) 78–79:219–35. 10.1016/j.matbio.2018.02.001 29425695

[24] Jackson DG. Leucocyte trafficking via the lymphatic vasculature- mechanisms and consequences. Front Immunol. (2019) 10:471. 10.3389/fimmu.2019.00471 30923528 PMC6426755

[25] Voisin MB Woodfin A Nourshargh S. Monocytes and neutrophils exhibit both distinct and common mechanisms in penetrating the vascular basement membrane *in vivo*. Arterioscler Thromb Vasc Biol. (2009) 29:1193–9. 10.1161/ATVBAHA.109.187450 19498176 PMC2712455

[26] Arokiasamy S Zakian C Dilliway J Wang W Nourshargh S Voisin MB. Endogenous TNFalpha orchestrates the trafficking of neutrophils into and within lymphatic vessels during acute inflammation. Sci Rep. (2017) 7:44189. 10.1038/srep44189 28287124 PMC5347029

[27] Finsterbusch M Voisin MB Beyrau M Williams TJ Nourshargh S. Neutrophils recruited by chemoattractants *in vivo* induce microvascular plasma protein leakage through secretion of TNF. J Exp Med. (2014) 211:1307–14. 10.1084/jem.20132413 24913232 PMC4076577

[28] Pflicke H Sixt M. Preformed portals facilitate dendritic cell entry into afferent lymphatic vessels. J Exp Med. (2009) 206:2925–35. 10.1084/jem.20091739 19995949 PMC2806476

[29] Champagne B Tremblay P Cantin A Pierre Y St. Proteolytic cleavage of ICAM-1 by human neutrophil elastase. J Immunol. (1998) 161:6398–405. 9834131

[30] Van Golen RF Van Gulik TM Heger M. Mechanistic overview of reactive species-induced degradation of the endothelial glycocalyx during hepatic ischemia/reperfusion injury. Free Radic Biol Med. (2012) 52:1382–402. 10.1016/j.freeradbiomed.2012.01.013 22326617

[31] Miao HQ Navarro E Patel S Sargent D Koo H Wan H. Cloning, expression, and purification of mouse heparanase. Protein Expr Purif. (2002) 26:425–31. 10.1016/S1046-5928(02)00558-2 12460766

[32] Vlodavsky I Iozzo RV Sanderson RD. Heparanase: multiple functions in inflammation, diabetes and atherosclerosis. Matrix Biol. (2013) 32:220–2. 10.1016/j.matbio.2013.03.001 23499526

[33] Mayfosh AJ Baschuk N Hulett MD. Leukocyte heparanase: a double-edged sword in tumor progression. Front Oncol. (2019) 9:331. 10.3389/fonc.2019.00331 31110966 PMC6501466

[34] Teijeira A Rouzaut A Melero I. Initial afferent lymphatic vessels controlling outbound leukocyte traffic from skin to lymph nodes. Front Immunol. (2013) 4:433. 10.3389/fimmu.2013.00433 24368908 PMC3856852

[35] Scruggs AK Cioffi EA Cioffi DL King JA Bauer NN. Lectin-Based characterization of vascular cell microparticle glycocalyx. PLoS ONE. (2015) 10:e0135533. 10.1371/journal.pone.0135533 26274589 PMC4537305

[36] Wang WC Cummings RD. The immobilized leukoagglutinin from the seeds of *Maackia amurensis* binds with high affinity to complex-type Asn-linked oligosaccharides containing terminal sialic acid-linked alpha-2,3 to penultimate galactose residues. J Biol Chem. (1988) 263:4576–85. 3350806

[37] Knibbs RN Goldstein IJ Ratcliffe RM Shibuya N. Characterization of the carbohydrate binding specificity of the leukoagglutinating lectin from *Maackia amurensis*. Comparison with other sialic acid-specific lectins. J Biol Chem. (1991) 266:83–8. 1985926

[38] Nicholls JM Bourne AJ Chen H Guan Y Peiris JS. Sialic acid receptor detection in the human respiratory tract: evidence for widespread distribution of potential binding sites for human and avian influenza viruses. Respir Res. (2007) 8:73. 10.1186/1465-9921-8-73 17961210 PMC2169242

[39] Khatua B Roy S Mandal C. Sialic acids siglec interaction: a unique strategy to circumvent innate immune response by pathogens. Indian J Med Res. (2013) 138:648–62. Available online at: http://www.ijmr.org.in/text.asp?2013/138/5/648/124646 24434319 PMC3928697

[40] Nightingale TD Frayne ME Clasper S Banerji S Jackson DG. A mechanism of sialylation functionally silences the hyaluronan receptor LYVE-1 in lymphatic endothelium. J Biol Chem. (2009) 284:3935–45. 10.1074/jbc.M805105200 19033446

[41] Ochoa-Alvarez JA Krishnan H Shen Y Acharya NK Han M Mcnulty DE. Plant lectin can target receptors containing sialic acid, exemplified by podoplanin, to inhibit transformed cell growth and migration. PLoS ONE. (2012) 7:e41845. 10.1371/journal.pone.0041845 22844530 PMC3402461

[42] Natunen S Lampinen M Suila H Ritamo I Pitkanen V Nairn AV. Metabolic glycoengineering of mesenchymal stromal cells with N-propanoylmannosamine. Glycobiology. (2013) 23:1004–12. 10.1093/glycob/cwt039 23708401 PMC3695754

[43] Proudfoot AEI Johnson Z Bonvin P Handel TM. Glycosaminoglycan interactions with chemokines add complexity to a complex system. Pharmaceuticals. (2017) 10:E70. 10.3390/ph10030070 28792472 PMC5620614

[44] Gouverneur M Spaan JA Pannekoek H Fontijn RD Vink H. Fluid shear stress stimulates incorporation of hyaluronan into endothelial cell glycocalyx. Am J Physiol Heart Circ Physiol. (2006) 290:H458–2. 10.1152/ajpheart.00592.2005 16126814

[45] Weber M Hauschild R Schwarz J Moussion C De Vries I Legler DF. Interstitial dendritic cell guidance by haptotactic chemokine gradients. Science. (2013) 339:328–32. 10.1126/science.1228456 23329049

[46] Johnson LA Banerji S Lawrance W Gileadi U Prota G Holder KA. Dendritic cells enter lymph vessels by hyaluronan-mediated docking to the endothelial receptor LYVE-1. Nat Immunol. (2017) 18:762–70. 10.1038/ni.3750 28504698

[47] Khan AI Kerfoot SM Heit B Liu L Andonegui G Ruffell B. Role of CD44 and hyaluronan in neutrophil recruitment. J Immunol. (2004) 173:7594–601. 10.4049/jimmunol.173.12.7594 15585887

[48] Mcdonald B Kubes P. Interactions between CD44 and Hyaluronan in Leukocyte Trafficking. Front Immunol. (2015) 6:68. 10.3389/fimmu.2015.00068 25741341 PMC4330908

[49] Gorlino CV Ranocchia RP Harman MF Garcia IA Crespo MI Moron G. Neutrophils exhibit differential requirements for homing molecules in their lymphatic and blood trafficking into draining lymph nodes. J Immunol. (2014) 193:1966–74. 10.4049/jimmunol.1301791 25015824

[50] Hampton HR Bailey J Tomura M Brink R Chtanova T. Microbe-dependent lymphatic migration of neutrophils modulates lymphocyte proliferation in lymph nodes. Nat Commun. (2015) 6:7139. 10.1038/ncomms8139 25972253 PMC4479041

[51] Chappell D Hofmann-Kiefer K Jacob M Rehm M Briegel J Welsch U. TNF-α induced shedding of the endothelial glycocalyx is prevented by hydrocortisone and antithrombin. Basic Res Cardiol. (2009) 104:78–89. 10.1007/s00395-008-0749-5 18836678

[52] Becker BF Chappell D Jacob M. Endothelial glycocalyx and coronary vascular permeability: the fringe benefit. Basic Res Cardiol. (2010) 105:687–701. 10.1007/s00395-010-0118-z 20859744

[53] Vanteeffelen JW Brands J Jansen C Spaan JA Vink H. Heparin impairs glycocalyx barrier properties and attenuates shear dependent vasodilation in mice. Hypertension. (2007) 50:261–7. 10.1161/HYPERTENSIONAHA.107.089250 17452501

[54] Chen G Wang D Vikramadithyan R Yagyu H Saxena U Pillarisetti S. Inflammatory cytokines and fatty acids regulate endothelial cell heparanase expression. Biochemistry. (2004) 43:4971–7. 10.1021/bi0356552 15109255

[55] Matzner Y Bar-Ner M Yahalom J Ishai-Michaeli R Fuks Z Vlodavsky I. Degradation of heparan sulfate in the subendothelial extracellular matrix by a readily released heparanase from human neutrophils. Possible role in invasion through basement membranes. J Clin Invest. (1985) 76:1306–13. 10.1172/JCI112104 2997275 PMC424062

[56] Komatsu N Waki M Sue M Tokuda C Kasaoka T Nakajima M. Heparanase expression in B16 melanoma cells and peripheral blood neutrophils before and after extravasation detected by novel anti-mouse heparanase monoclonal antibodies. J Immunol Methods. (2008) 331:82–93. 10.1016/j.jim.2007.11.014 18162185

[57] Lukasz A Hillgruber C Oberleithner H Kusche-Vihrog K Pavenstadt H Rovas A. Endothelial glycocalyx breakdown is mediated by angiopoietin-2. Cardiovasc Res. (2017) 113:671–80. 10.1093/cvr/cvx023 28453727

[58] Curry FE Adamson RH. Endothelial glycocalyx: permeability barrier and mechanosensor. Ann Biomed Eng. (2012) 40:828–39. 10.1007/s10439-011-0429-8 22009311 PMC5042904

[59] Bridenbaugh EA Nizamutdinova IT Jupiter D Nagai T Thangaswamy S Chatterjee V. Lymphatic muscle cells in rat mesenteric lymphatic vessels of various ages. Lymphat Res Biol. (2013) 11:35–42. 10.1089/lrb.2012.0025 23531183 PMC3609606

[60] Hirakawa S Detmar M Karaman S. Lymphatics in nanophysiology. Adv Drug Deliv Rev. (2014) 74:12–8. 10.1016/j.addr.2014.01.011 24524932

[61] Kerjaschki D. The lymphatic vasculature revisited. J Clin Invest. (2014) 124:874–7. 10.1172/JCI74854 24590271 PMC3938252

