# Supplementary material for: Heparanase-Dependent Remodeling of Initial Lymphatic Glycocalyx Regulates Tissue-Fluid Drainage During Acute Inflammation in vivo
Source: Front Immunol. 2019 Oct 4;10:2316. doi: 10.3389/fimmu.2019.02316 (PMC6787176; doi:10.3389/fimmu.2019.02316)
Supplement: Supplementary file 1 [file Presentation_1.pdf]

Supplementary information

"Heparanase-Dependent Remodelling of Initial Lymphatic Glycocalyx Regulates Tissue-fluid Drainage during Acute Inflammation" by Arokiasamy S. et al.

<u>Content:</u>

Supplementary Figures 1-6

Supplementary Figure 1

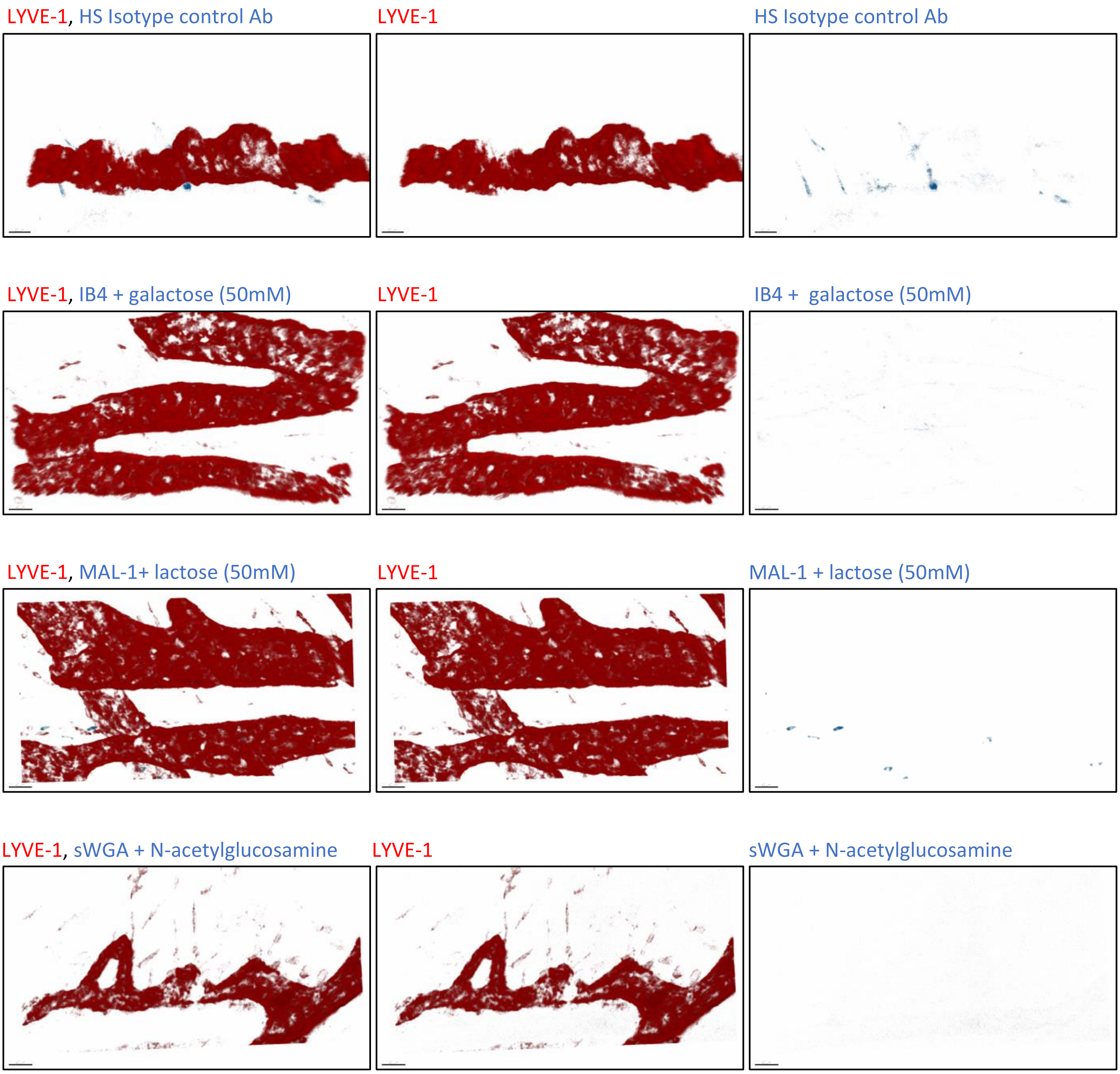

Supplementary Figure 1: Staining specificity of glycocalyx moieties in whole-mount cremaster muscles as observed by confocal microscopy. Mouse received an i.s. injection for 90-120 mins of fluorescently labelled LYVE-1 and mouse Ig control antibody (control Ab for HS), or lectin pre-incubated with their inhibitory carbohydrate moieties, i.e. galactose for IB4, lactose for MAL1 and N-Acetylglucosamine for sWGA. The pictures are representative 3D-reconstructed confocal images of a region of the cremaster muscle showing the channel for LYVE-1 and inhibited lectin/Isotype control Ab. Bar = 40µm. Images are representative pictures from 3 animals.

Supplementary Figure 2

A

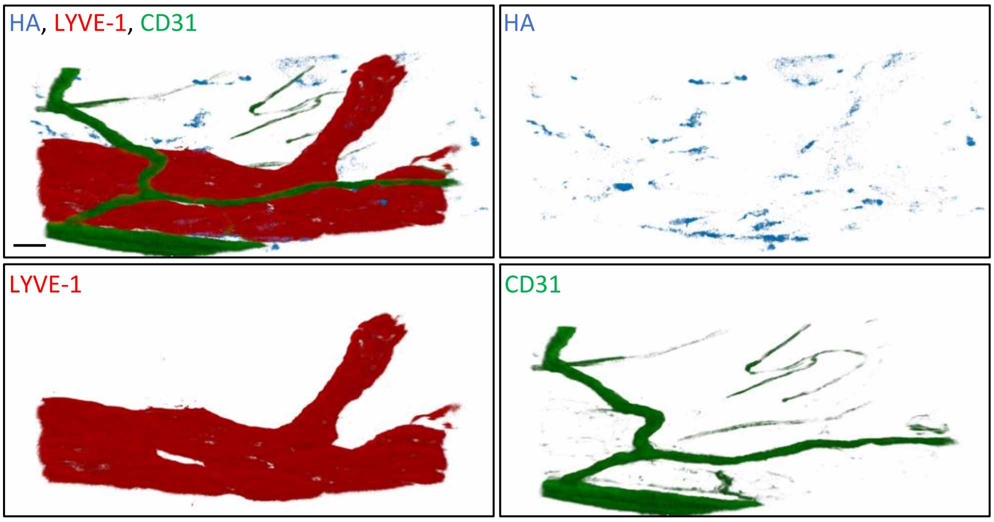

B

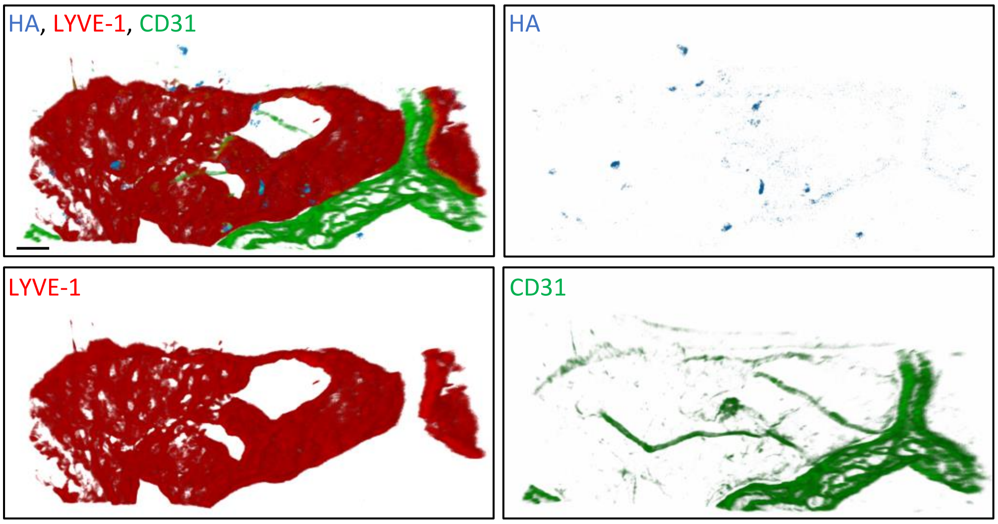

Supplementary Figure 2: Hyaluronan detection in the cremaster muscles as observed by confocal microscopy. Mouse received an i.s. injection of anti-hyaluronic acid (HA), anti-LYVE-1 and anti-CD31 injection for 90 mins to reveal the hyaluronan (HA), the lymphatic and blood vasculatures, respectively, prior to the visualisation of the samples by confocal microscopy. The pictures are representative 3D-reconstructed confocal images of a region of an unstimulated (PBS) (**A**) or TNF (16hrs)-stimulated (**B**) cremaster muscle showing that hyaluronan (blue) is not associated with cremaster lymphatic (LYVE-1, red) or blood (CD31, green) vasculatures but with discrete cellular structures present within the interstitial tissue. Bar = 40μm. Images are representative pictures from at least 5 vessels/animals from 5 animals.

Supplementary Figure 3

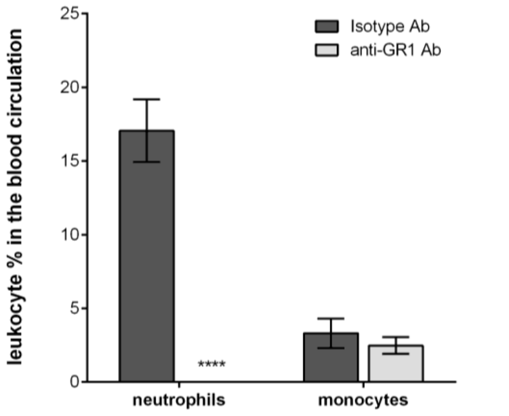

Supplementary Figure 3: Effect of an anti-GR1 Ab-induced neutrophil depletion on leukocyte populations in the blood. Mice received 3 consecutive i.p. injections of 25µg of anti-GR1 Ab (clone RB6-8C5) per mouse per day prior to the induction of the inflammatory reaction. A blood sample from the tail was collected prior to the injection of the inflammatory mediator and neutrophil (Ly6G+) and monocytes (CD115+) populations were analysed by flow cytometry. N = at least 6 mice per group.

Supplementary Figure 4

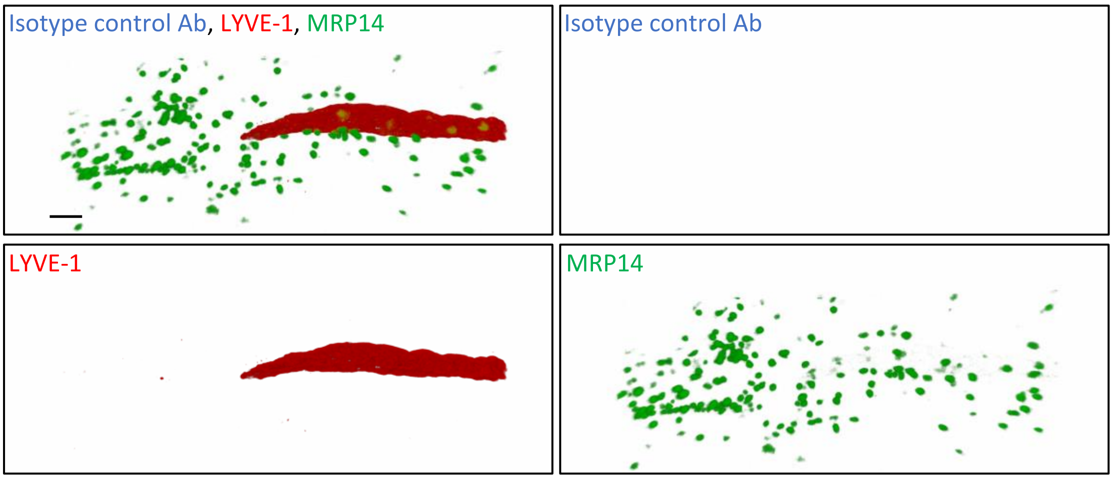

Supplementary Figure 1: Staining specificity of Heparanase I immunostaining in whole-mount cremaster muscles as observed by confocal microscopy. Mouse cremaster muscles were stimulated with 300ng of TNF. At the end of the inflammatory period (16hrs), tissue were removed, fixed/permeabilised and immunostained with of fluorescently labelled LYVE-1 (lymphatic vessels), MRP14 (neutrophils) and a rabbit Ig control antibody (control Ab for Heparanase I). The pictures are representative 3D-reconstructed confocal images of a region of the cremaster muscle showing the absence of non-specific staining in the tissue. Bar = 50µm. Images are representative pictures from 3 animals.

Supplementary Figure 5

A **B**

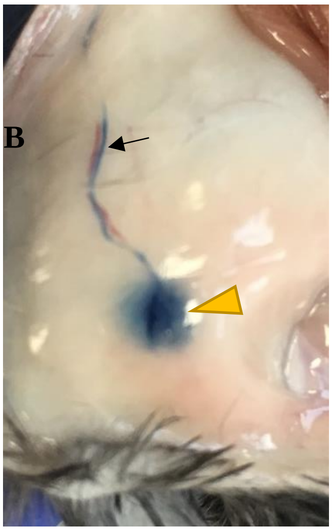

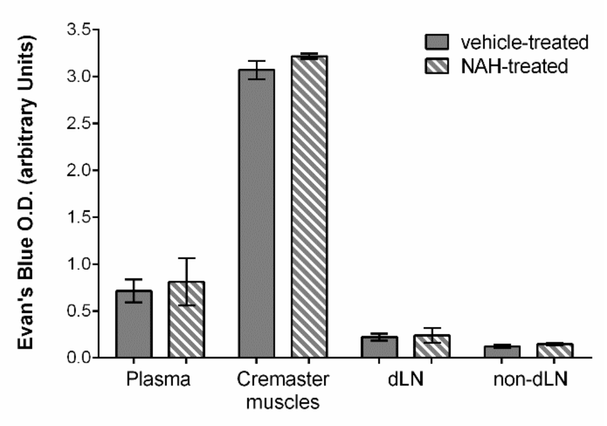

Supplementary Figure 5: Effect of a non-anticoagulant heparanase inhibitor (NAH) on lymphatic drainage in uninflamed conditions. PBS was administrated intrascrotally. Three hours later, mice received an i.s. injection of 50µg of non-anticoagulant heparanase inhibitor N-desulfated/re-N-acetylated heparin (NAH) or vehicle. Twenty minutes before the end of the first injection (i.e. 16 hrs post PBS injection), mice received an i.s. injection of 1% Evans Blue. Animals were then sacrificed, their plasma, cremaster tissues, draining and non-draining lymph nodes were collected and prepared for spectrophotometric analysis of Evans Blue content. (**A**) Representative photographic image of an inguinal lymph node (dLN) draining the cremaster muscle of a mice injected i.s. with 1% Evans Blue for 20 min. The yellow arrowhead shows the position of the dLN and the plain black arrow shows the dLN-associated efferent lymphatic venule (and running alongside a blood vessel), both containing Evans Blue. (**B**) Quantification of the Evans Blue content in the mouse cremaster in mice treated with or without NAH. (N= 3-6 mice per group).

Supplementary Figure 6

A

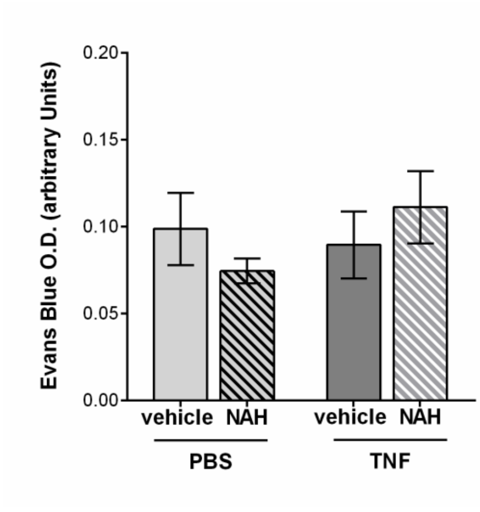

Supplementary Figure 6: Effect of a non-anticoagulant heparanase inhibitor (NAH) on blood vascular leakage. Blood vascular leakage and lymphatic drainage was assessed using the Miles Assay. Briefly, mice were first injected i.s. with TNF (30ng) or PBS. Three hours later, mice received an i.s. injection of 50µg of non-anticoagulant heparanase inhibitor N-desulfated/re-N-acetylated heparin (NAH) or vehicle. Two hours before the end of the first i.s. injection (i.e. 14 hrs post TNF/PBS injection), mice received an intravenous injection of injection of 0.5% Evans Blue (5µL/g). At the end of the inflammatory period, animals were sacrificed and their cremaster tissues were collected and prepared for spectrophotometric analysis of Evans Blue content. (N= 4-6 mice per group).
